# Radiofluorination and biological evaluation of *N*-aryl-oxadiazolyl-propionamides as potential radioligands for PET imaging of cannabinoid CB_2_ receptors

**DOI:** 10.1186/2191-2858-3-11

**Published:** 2013-09-24

**Authors:** Rodrigo Teodoro, Rareş-Petru Moldovan, Corinna Lueg, Robert Günther, Cornelius K Donat, Friedrich-Alexander Ludwig, Steffen Fischer, Winnie Deuther-Conrad, Bernhard Wünsch, Peter Brust

**Affiliations:** 1 Department of Neuroradiopharmaceuticals, Institute of Radiopharmaceutical Cancer Research, Research Site Leipzig, Helmholtz-Zentrum Dresden-Rossendorf e.V., Permoserstr 15, 04318 Leipzig, Germany; 2 Department of Pharmaceutical and Medicinal Chemistry, University of Münster, Corrensstraße 58-62, 48149 Münster, Germany

**Keywords:** Blood–brain barrier, Cannabinoid receptors, ^18^F labelling, Molecular imaging, Positron emission tomography

## Abstract

**Background:**

The level of expression of cannabinoid receptor type 2 (CB_2_R) in healthy and diseased brain has not been fully elucidated. Therefore, there is a growing interest to assess the regional expression of CB_2_R in the brain. Positron emission tomography (PET) is an imaging technique, which allows quantitative monitoring of very low amounts of radiolabelled compounds in living organisms at high temporal and spatial resolution and, thus, has been widely used as a diagnostic tool in nuclear medicine. Here, we report on the radiofluorination of *N*-aryl-oxadiazolyl-propionamides at two different positions in the lead structure and on the biological evaluation of the potential of the two tracers [^18^F]**1** and [^18^F]**2** as CB_2_ receptor PET imaging agents.

**Results:**

High binding affinity and specificity towards CB_2_ receptors of the lead structure remained unaffected by the structural changes such as the insertion of the aliphatic and aromatic fluorine in the selected labelling sites of **1** and **2**. Aliphatic and aromatic radiofluorinations were optimized, and [^18^F]**1** and [^18^F]**2** were achieved in radiochemical yields of ≥30% with radiochemical purities of ≥98% and specific activities of 250 to 450 GBq/μmol. Organ distribution studies in female CD1 mice revealed that both radiotracers cross the blood–brain barrier (BBB) but undergo strong peripheral metabolism. At 30 min after injection, unmetabolized [^18^F]**1** and [^18^F]**2** accounted for 60% and 2% as well as 68% and 88% of the total activity in the plasma and brain, respectively. The main radiometabolite of [^18^F]**2** could be identified as the free acid [^18^F]**10**, which has no affinity towards the CB_1_ and CB_2_ receptors but can cross the BBB.

**Conclusions:**

*N*-aryl-oxadiazolyl-propionamides can successfully be radiolabelled with ^18^F at different positions. Fluorine substitution at these positions did not affect affinity and specificity towards CB_2_R. Despite a promising *in vitro* behavior, a rather rapid peripheral metabolism of [^18^F]**1** and [^18^F]**2** in mice and the generation of brain permeable radiometabolites hamper the application of these radiotracers *in vivo*. However, it is expected that future synthetic modification aiming at a replacement of metabolically susceptible structural elements of [^18^F]**1** and [^18^F]**2** will help to elucidate the potential of this class of compounds for CB_2_R PET studies.

## Background

Cannabinoid receptors (CBR) belong to the superfamily of G protein-coupled receptors (GPCR) and are involved in various physiological processes. Besides the CBR identified so far, the cannabinoid receptor type 1 (CB_1_R) [1] and type 2 (CB_2_R) [2], another GPCR natively interacting with endogenous cannabinoids (GPR55) has been proposed as a third type of CBR [3,4]. While the CB_1_R is primarily expressed in the central nervous system, the CB_2_R is predominantly located in the periphery, especially in tissues related to the immune system. In pathological conditions, the up-regulation of CB_2_R expression is mostly associated with inflammatory processes [5,6], neuropathic pain [7-9], Alzheimer’s disease, and amyotrophic lateral sclerosis (ALS) [10-13]. It has also been shown that the activation of CB_2_R is connected with the induction of apoptosis in several cancer cell lines [14-17]. However, CB_2_R are also expressed in healthy brain under physiological conditions at very low expression levels [18,19]. Therefore, the non-invasive quantification of CB_2_R in the brain, possibly in general by application of highly sensitive imaging techniques such as positron emission tomography (PET), requires the availability of radioligands binding with high affinity and high specificity towards CB_2_R [20]. Apart from the numerous specific pharmacologically relevant ligands of CB_2_R reported so far (see [21,22]), only a limited number have been applied for the development of PET radiotracers for imaging of CB_2_R [23-29]. As we reported earlier, the increase of brain uptake and metabolic stability and decrease of non-specific binding remained as a challenge for further development [29]. Even a follow-up study on [^11^C]AZD1940 in non-human primate with PET confirmed a relatively low CNS exposure of this radioligand [28]. Mu et al. reported on *N*-(1-adamantyl)-8-methoxy-4-oxo-1-phenyl-1,4-dihydroquinoline-3-carboxamide as a ^11^C-labelled PET probe for imaging of CB_2_R [30]. Although the study showed a rather low brain uptake, and two blocking experiments with GW4058233 to demonstrate the specificity of brain uptake were not conclusive, the potential of the compound for PET imaging of amyotrophic lateral sclerosis has been proposed.

Further, a whole-body biodistribution and radiation dosimetry study of the CB_2_R ligand [^11^C]NE40 has been performed in healthy subjects [31]. It showed the expected biodistribution being compatible with lymphoid tissue (spleen) uptake and an appropriate uptake and kinetics in the brain. This underscores the potential of this tracer for application in central and peripheral inflammation imaging. Despite this considerable progress in CB_2_R PET imaging, a suitable ligand [32] radiolabelled with the advantageous longer-lived isotope ^18^F is still missing.

Recently, we reported on the synthesis, radiofluorination, and first biological investigation of the *N*-aryl-oxadiazolyl-propionamide [^18^F]**2** as a potential radioligand for the PET imaging of CB_2_R [29]. Although the initial radiofluorination approach using a nitro precursor proved to be unsatisfactory (yields ≤ 3%), the promising biological findings encouraged us to revise and upgrade the radiosynthesis of [^18^F]**2** and to perform a more detailed biological evaluation.

## Methods

**Figure 1:**
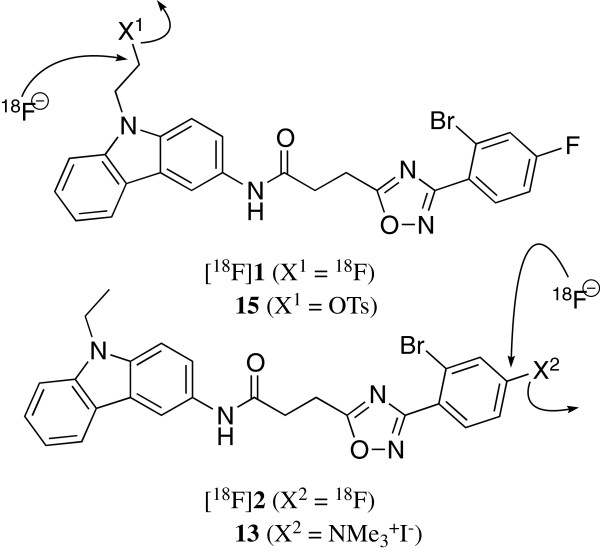
**Aliphatic and aromatic radiofluorination of *N*-aryl-oxadiazolyl-propionamides.**

We describe an improved synthesis of [^18^F]**2** using a trimethylammonium precursor for the radiolabelling at the aromatic site of the lead structure (Figure 1, compound **13**, *X*^2^ = NMe_3_^+^I). In parallel, we explored the aliphatic radiofluorination at the carbazole *N*-alkyl chain (Figure 1, compound **15**, *X*^1^ = OTs). The labelling of the lead compound at two different sites opens up the possibility to investigate the dependence of affinity, biodistribution and metabolism of these radiotracers on the site of radiolabelling.

## Results and discussion

### Synthesis of *N*-arylamide oxadiazoles: precursors for radiochemistry and reference compounds

**Scheme 1:**
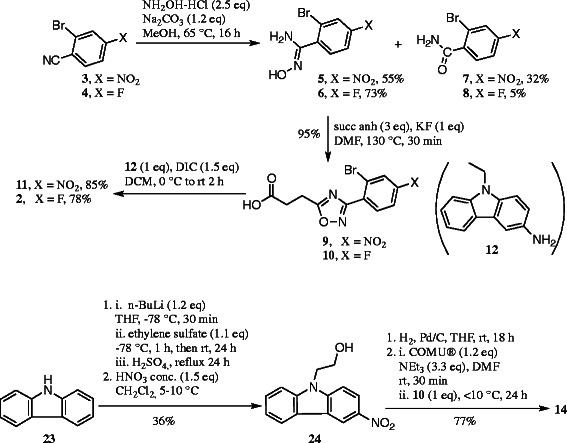
**Synthesis of the *N*-aryl-oxadiazolyl-propionamide derivatives 11, 2, and 14.** NH_2_OH-HCl, hydroxylamine hydrochloride; succ anh, succinic anhydride; DIC, *N*,*N*′-diisopropylcarbodiimide; COMU®, (1-Cyano-2-ethoxy-2-oxoethylidenaminooxy) dimethylamino-morpholino-carbenium hexafluorophosphate.

In a previous study, we have described the synthesis of >20 fluorinated *N*-arylamide oxadiazoles including labelling precursors for radiochemistry [29]. Here we describe a modified and improved route to obtain novel derivatives for high-yield radiochemistry. In brief, treatment of the nitriles **3** and **4** with excess of hydroxylamine hydrochloride under alkaline conditions delivered the (Z)-amidoximes **5** and **6** in 55% and 73% yield together with amides **7** and **8** (Scheme 1), which have been separated by column chromatography on silica. The Z configuration of the amidoximes was determined by two-dimensional nuclear magnetic resonance (NMR) spectroscopy. Treatment of the amidoximes **5** and **6** with excess of succinic anhydride delivered the acids **9** and **10** in a nearly quantitative manner. The *N*-aryl-oxadiazolyl-propionamides **11** and **2** were obtained in high yields (85% and 78%) by using *N*, *N*′-diisopropylcarbodiimide (DIC) as coupling reagent [33].

**Scheme 2:**
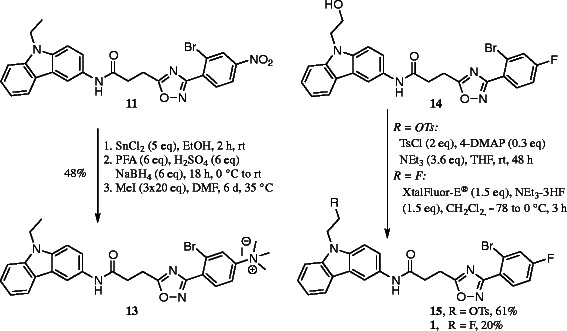
**Synthesis of precursors 13 and 15 and reference compound 1.** SnCl_2_, tin(II) chloride; PFA, paraformaldehyde; MeI, methyl iodide; 4-DMAP, 4-dimethylaminopyridine; XtalFluor-E®, diethylaminodifluorosulfonium tetrafluoroborate; NEt_3_-3HF, triethylamine trihydrofluoride.

For the aromatic radiofluorination, trimethylammonium iodide has been used as a leaving group. Although methyl iodide (MeI) has a very high nucleophilicity, the choice of I^−^ as a counter ion speeds up the displacement of the trimethylammonium salt in an aromatic ring during radiofluorination under mild conditions [34], thus lowering the amount of potential formation of non-radioactive and radioactive by-products. The trimethylammonium salt **13** was synthesized from the nitro derivative **11** by reduction, reductive methylation with paraformaldehyde and NaBH_4_[35], and quaternization by employing large excess of MeI (Scheme 2) [36].

The ethanol derivative **14** was synthesized in four steps and 28% overall yield starting from the commercially available carbazole (**23**). The synthesis started with deprotonation of carbazole with *n*-BuLi, reaction of the carbazolyl anion with ethylene sulfate, and subsequent hydrolysis with dilute H_2_SO_4_ to afford the hydroxyethyl derivative **25** (see ‘Experimental’ section). The crystalline cyclic ethylene sulfate represents a non-toxic but reactive alternative to the gaseous and toxic oxirane. However, there are only few examples for the introduction of a 2-hydroxyethyl group using ethylene sulfate. Nitration of the hydroxyethylcarbazole **25** was performed with concentrated nitric acid at 5°C to 10°C to give the 3-nitro carbazole derivative [37]**24** in 54% yield (Scheme 1). Reduction of the nitrocarbazole **24** with H_2_ in the presence of Pd/C provided the primary aromatic amine **26**, which was precipitated as HCl salt. The final coupling of the carbazolamine **26** with the propionic acid **10** was induced by COMU® providing the amide **14** (Scheme 1).

The fluoroethyl reference compound **1** was prepared by treatment of **14** with the fluorinating agent diethylaminodifluorosulfonium tetrafluoroborate (XtalFluor-E®) (Scheme 2). In order to obtain the precursor for the radiosynthesis, the alcohol **14** was transformed into the tosylate **15** since the tosyloxy moiety represents a good leaving group for the nucleophilic substitution with [^18^F]fluoride.

**Scheme 3:**
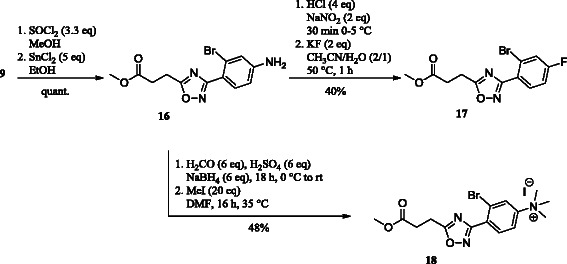
Synthesis of the trimethylammonium salt 18 and reference compound 17.

For investigations regarding the main metabolite [^18^F]**10**, the trimethylammonium salt **18** and reference compound **17** were synthesized. Intermediate **16** was obtained by acid protection followed by reduction from **9**. To synthesize the reference compound **17**, Sandmeyer reaction has been applied [38]. The use of tetrafluoroborate as fluorinating reagent led to the formation of a complex reaction mixture whereas the use of KF delivered **17** in 40% yields. Alternatively, **17** could also be obtained from **10** (not shown in Scheme 3, see ‘Experimental’ section) in improved yield. The trimethylammonium salt **18** was obtained as described above in 48% over two steps.

### Radiochemistry

**Scheme 4:**
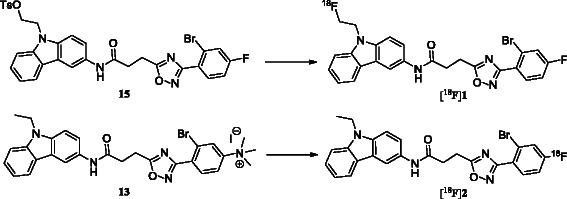
**Radiosynthesis of [**^**18**^**F]1 and [**^**18**^**F]2.** K[^18^F]F-K_2.2.2_-carbonate complex (5.6 mg, 15 μmol), 2 mg (**15** or **13**), MeCN, 82°C, 10 min.

**Figure 2:**
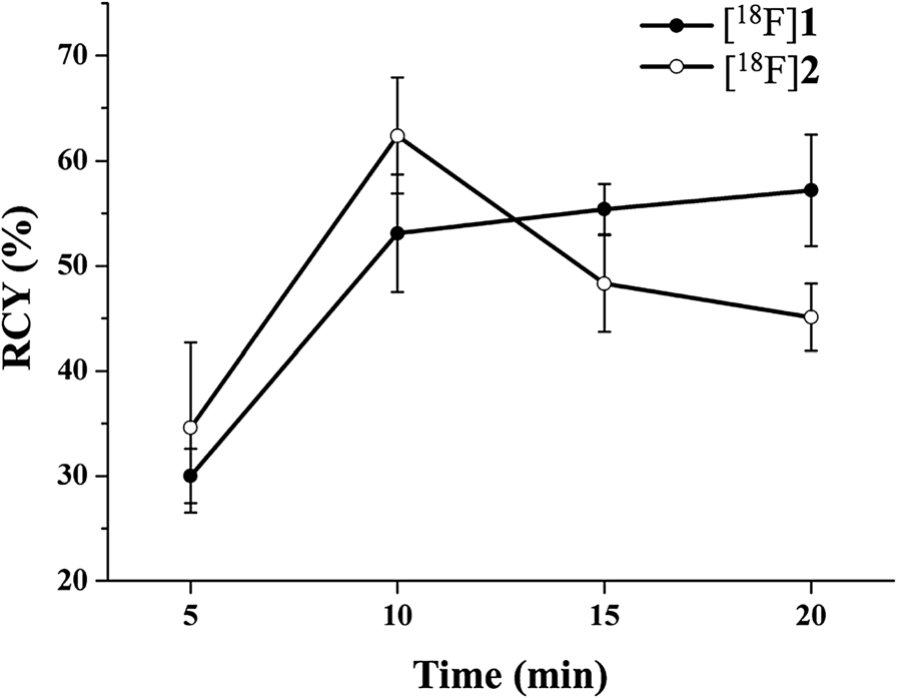
**Time-yield curves of the aliphatic ([**^**18**^**F]1) and aromatic ([**^**18**^**F]2) radiolabellings in MeCN up to 20 min.**

Reaction conditions, purification, and formulation procedures were optimized to achieve a high radiochemical yield (RCY) and a high radiochemical purity in a short synthesis time. Aliphatic and aromatic radiolabellings were carried out at 82°C in MeCN under no-carrier-added (NCA) conditions (Scheme 4). High labelling yields (Figure 2) were obtained using 2 mg of precursor. For [^18^F]**1**, the optimal conversion of the tosylate **15** was achieved after 10 min (53 ± 6%, *n* = 12). For [^18^F]**2**, a maximum was reached after 10 min (63 ± 5%, *n* = 7), along with a steady loss of product (51 ± 6%, 15 min) thereafter, probably due to the decomposition of the trimethylammonium salts under prolonged heating [39]. Altogether, evaluating the current results of labelling of [^18^F]**2**, a great improvement was obtained by using the herein described new trimethylammonium precursor **13** instead of the formerly applied nitro compound **11** (see Scheme 4) where a radiochemical yield of only 3% has been achieved [29].

**Figure 3:**
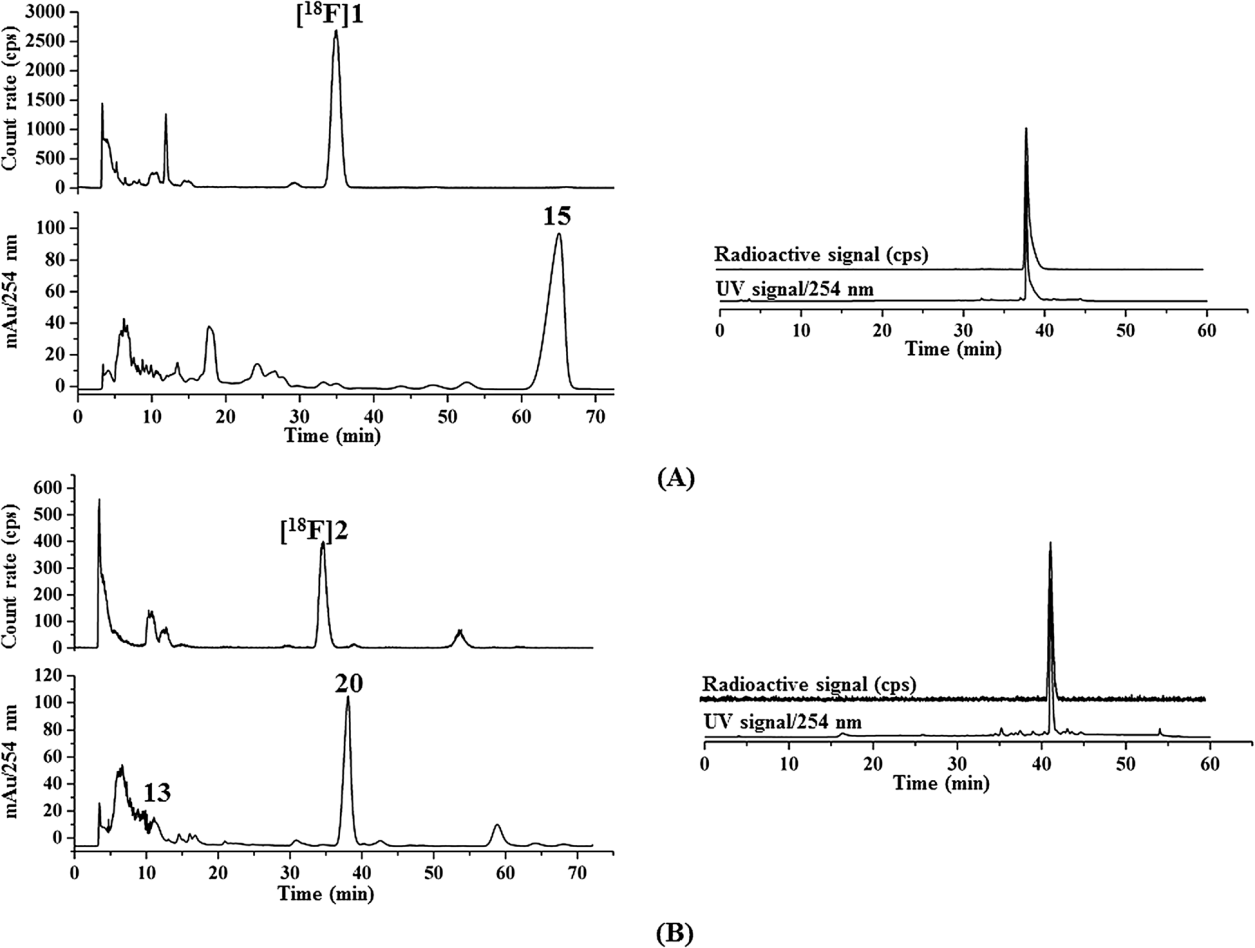
**High-performance liquid chromatography.** Isocratic semi-preparative RP-HPLC chromatograms (left) and the analytical HPLC chromatograms (right) of the radiotracers spiked with the respective reference compounds. **(A)** [^18^F]**1** (*t*_R_ = 35.3 min), eluent: 58% MeCN/10 mM NH_4_OAc aq. on a Reprosil Gold C18, 10 μm, 150 × 4.6 mm; flow rate: 2.5 mL/min. **(B)** [^18^F]**2** (*t*_R_ = 35.6 min), eluent: 60% MeCN/20 mM NH_4_OAc aq. on a Multospher 120 RP-18 AQ, 5 μm, 150 × 10 mm; flow rate: 2.0 mL/min. The precursors for radiolabelling (**13** and **15**) and compound **20** (see ‘Experimental’ section) were also identified by analytical HPLC on a Multospher 120 RP 18 AQ column, 5 μm, 250 × 4.6 mm; solvents: A: 10% MeCN/20 mM NH_4_OAc, B: 90% MeCN/20 mM NH_4_OAc. Gradient elution(A%): 0 to 10 min, 100%; 10 to 40 min, gradient from 100% to 0%; 40 to 45 min, 0%; 45 to 50 min, 100% at a flow rate of 1 mL/min.

Reaction mixtures were separated by isocratic semi-preparative HPLC, fractions were collected, and the identities confirmed by spiked analytical HPLC samples with the respective reference compounds of [^18^F]**1** and [^18^F]**2** (Figure 3). Final purification was performed by solid phase extraction (SPE) using Sep-Pack cartridges, and for biological investigations, the products were formulated in isotonic saline containing 10% EtOH. The radiotracers were produced with 30% to 35% RCY, high radiochemical purities (≥98%), and high specific activities (250 to 450 GBq/μmol, *n* = 4).

### *In vitro* stability and logD determination

Investigations on the *in vitro* stability of [^18^F]**1** and [^18^F]**2** were performed prior to the experimental determination of logD values. [^18^F]**1** exhibited a slight tendency to de-fluorinate at 40°C in Tris–HCl, EtOH, 0.9% NaCl, and Dulbecco’s phosphate buffer with 89%, 93%, 95%, and 94% of intact tracer after 30 min of incubation and no further decrease up to 90 min. [^18^F]**2** showed a very high stability with ≥98% of intact tracer at incubation in all selected media up to 90 min. Radio-thin-layer chromatography (TLC) and radio-HPLC analyses were in good agreement, and no further radioactive degradation products were observed.

Regarding the HPLC-based logD determination, differences were noticed between isocratic and gradient elutions using the same column. However, as compiled in Table 1, the logD values using Reprosil-Pur C18 AQ column under gradient conditions are in good agreement with those found with Prodigy 5 μm C8 column.

**Table 1:** Experimental logD coefficients of compounds 1 and 2

**Compound**	**Reposil-Pur C18-AQ**^**a**^		**Prodigy 5 μ C8**^**a**^
	**Isocratic**	**Gradient**	**Isocratic**
1	4.21 ± 0.32	3.82 ± 0.10	3.86 ± 0.33
2	4.87 ± 0.36	4.35 ± 0.11	4.40 ± 0.35

^a^Experiments performed with the respective reference compounds (*n* = 3 to 6).

### Receptor affinity

The substitution of the ethyl chain of the carbazole moiety in **2** with a fluorine-ethyl chain in **1** had little effect on target affinity and specificity as reflected by comparable *K*_i_ values for the CB_2_R (**1**, *K*_i_ 2.32 ± 2.12 nM; **2**, *K*_i_ 4.27 ± 3.03 nM) [29]. Based on the data published by Cheng et al. [40] (JCPDS 42–44), an agonistic profile of **1** and **2** can be assumed. Both compounds possess no affinity towards the CB_1_R (*K*_i_ > 1 μM). By contrast, one major brain-penetrating metabolite, compound **10,** shows almost no binding towards the CBRs (*K*_i CB1R_ > 1 μM, *K*_i CB2R_ > 1 μM). In accordance with the *ex vivo* autoradiographic studies on [^18^F]**2** in spleen tissue slices [29], compound [^18^F]**1** targets CB_2_R *in vivo* too. The autoradiograms are shown in Figure S1 of Additional file 1.

### Organ distribution of [^18^F]1 and [^18^F]2 in mice

The biodistribution of compounds [^18^F]**1** and [^18^F]**2** in percentage of injected dose per gram (% ID/g) at 5, 30, and 60 min post injection (p.i.) is compiled in Tables 2 and 3. For both radiotracers, the highest uptake was detected at 5 min p.i. in the spleen. Comparable kinetics were observed in the brain and most other organs such as the lung, liver, kidney, thymus, and adrenals after administration of [^18^F]**1** and [^18^F]**2**, respectively. Both compounds showed a high gastrointestinal excretion as reflected by the constant increase of activity uptake in the small intestine. A constantly low uptake of activity in the femur indicates no confounding defluorination during the experiment.

**Table 2:** **Radioactivity uptake in major organs of female CD1mice after intravenous administration of 300 to 400 kBq [**^**18**^**F]1**

**Organ**	**Control (% ID/g)**			**Blocking (% ID/g)**
	**5 min**^**a**^	**30 min**^**b**^	**60 min**^**b**^	**60 min**^**c**^
Blood	1.94 ± 0.44	1.44 ± 0.41	1.94 ± 0.69	1.43 ± 0.34
Plasma	2.52 ± 0.58	1.88 ± 0.58	2.50 ± 0.87	1.87 ± 0.41
Brain	1.31 ± 0.24	0.76 ± 0.22	0.92 ± 0.27	0.66 ± 0.11
Heart	3.39 ± 0.45	1.43 ± 0.47	1.66 ± 0.37	1.49 ± 0.29
Lung	3.68 ± 0.62	1.65 ± 0.70	1.77 ± 0.74	1.47 ± 0.29
Stomach	2.05 ± 0.33	1.23 ± 0.36	2.70 ± 1.47	1.84 ± 1.28
Small intestine	4.36 ± 2.09	10.51 ± 8.38	22.03 ± 8.32	19.39 ± 5.53
Large intestine	1.91 ± 2.66	3.24 ± 5.61	1.54 ± 0.42	1.63 ± 0.64
Liver	11.00 ± 1.04	4.38 ± 1.19	5.29 ± 1.22	4.22 ± 0.96
Kidney	13.40 ± 5.66	2.68 ± 0.37	1.65 ± 0.49	1.74 ± 0.48
Bladder	2.23 ± 1.39	1.65 ± 0.43	3.27 ± 1.94	5.07 ± 3.84
Spleen	3.44 ± 0.77	1.46 ± 0.56	1.52 ± 0.62	0.98 ± 0.21
Thymus	3.30 ± 0.58	1.57 ± 0.80	1.90 ± 0.98	1.50 ± 0.35
Pancreas	4.97 ± 0.62	1.50 ± 0.74	1.13 ± 0.11	1.03 ± 0.26
Adrenals	4.16 ± 6.23	3.31 ± 0.99	3.16 ± 1.25	3.00 ± 0.64
Gonads	1.06 ± 0.94	1.02 ± 0.26	1.33 ± 0.50	1.19 ± 0.30
Muscle	2.10 ± 0.33	1.02 ± 0.50	0.89 ± 0.19	0.93 ± 0.23
Femur	2.00 ± 0.38	1.37 ± 0.50	1.84 ± 0.35	1.49 ± 0.45

Blocking was performed with pre-administration of SR144528 (3 mg/kg i.p.). Values are mean ± SD. ^a^*n* = 4, ^b^*n* = 5, ^c^*n* = 6.

**Table 3:** **Radioactivity uptake in major organs of female CD1 mice after intravenous administration of 300 to 400 kBq [**^**18**^**F]2**

**Organ**	**Control (%ID/g)**			**Blocking (%ID/g)**
	**5 min**^**a**^	**30 min**^**b**^	**60 min**^**a**^	**60 min**^**a**^
Blood	2.29 ± 0.48	3.42 ± 0.39	2.50 ± 0.42	3.40 ± 0.21
Plasma	3.87 ± 0.90	6.82 ± 1.19	5.22 ± 0.73	5.78 ± 0.07
Brain	0.62 ± 0.22	0.86 ± 0.34	0.26 ± 0.07	0.28 ± 0.03
Heart	3.42 ± 0.57	2.32 ± 0.24	1.45 ± 0.20	1.53 ± 0.13
Lung	4.62 ± 0.21	3.36 ± 0.02	2.52 ± 0.60	2.31 ± 0.19
Stomach	1.57 ± 1.18	2.09 ± 0.81	2.21 ± 0.43	3.24 ± 2.10
Small intestine	3.86 ± 2.64	20.78 ± 3.28	27.31 ± 2.44	29.64 ± 5.10
Large intestine	0.46 ± 0.06	1.25 ± 0.30	1.86 ± 0.36	1.78 ± 0.23
Liver	31.31 ± 9.91	8.84 ± 0.83	6.27 ± 0.68	10.51 ± 1.10
Kidney	5.86 ± 0.96	5.90 ± 0.15	3.96 ± 1.25	4.91 ± 1.51
Bladder	1.01 ± 0.29	2.02 ± 0.39	1.45 ± 0.57	1.77 ± 0.16
Spleen	10.42 ± 7.67	1.34 ± 0.10	0.68 ± 0.11	0.74 ± 0.10
Thymus	2.30 ± 1.06	1.96 ± 0.22	1.04 ± 0.08	1.77 ± 0.14
Pancreas	2.04 ± 0.59	1.71 ± 0.15	0.82 ± 0.21	1.02 ± 0.11
Adrenals	7.67 ± 2.18	5.38 ± 0.61	2.44 ± 0.72	3.34 ± 0.15
Gonads	1.14 ± 0.32	2.08 ± 0.03	2.29 ± 0.57	1.97 ± 0.39
Muscle	1.00 ± 0.30	0.92 ± 0.15	0.70 ± 0.27	0.70 ± 0.06
Femur	1.63 ± 0.93	1.10 ± 0.03	0.58 ± 0.11	0.61 ± 0.09

Blocking was performed with pre-administration of SR144528 (3 mg/kg i.p.). Values are mean ± SD. ^a^*n* = 3, ^b^*n* = 2.

The highest accumulation of activity was found in the kidneys and liver for both compounds. While the activity uptake is higher in the kidneys for [^18^F]**1**, the values for [^18^F]**2** are higher in the liver, indicating different excretion patterns.

Blocking experiments were performed with pre-administration of the CB_2_R-specific inverse agonist SR144528. At 60 min, the animals were sacrificed and the percentage of injected dose per gram of activity uptake was calculated for the various organs. No significant reduction of percentage of injected dose per gram could be observed for both compounds in any of the organs investigated including CB_2_R-expressing organs (details are compiled in Additional file 1: Table S1). For [^18^F]**2**, the pre-administration of SR144528 led to an increase of percentage of injected dose per gram in some organs, which might be related to metabolic processes.

**Figure 4:**
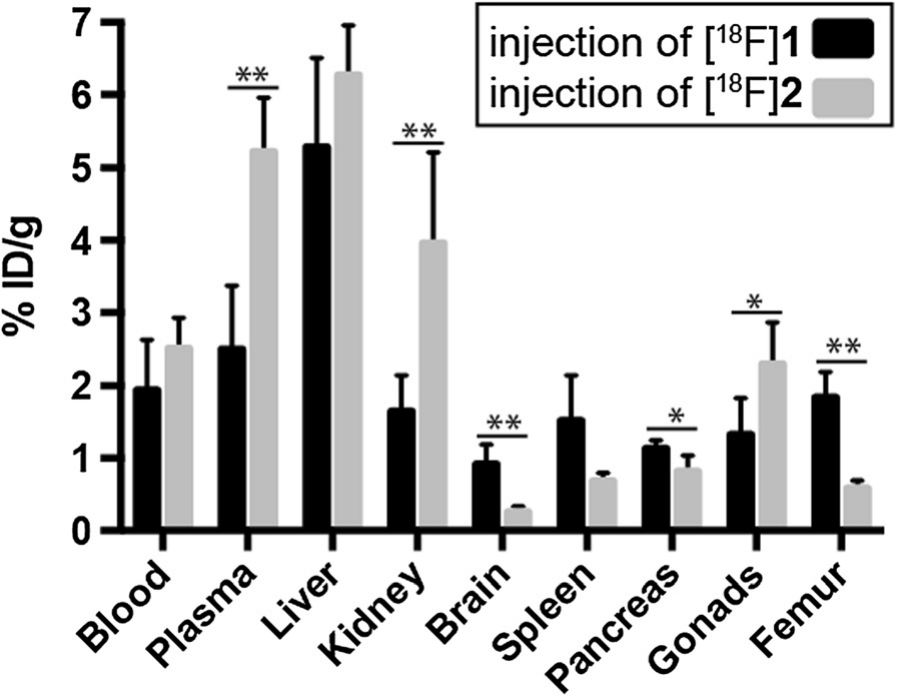
**Distribution of radioactivity at 60 min in selected organs after administration of the tracers [**^**18**^**F]1 and [**^**18**^**F]2.** Values are mean ± SD. Significant differences are indicated (^*^p < 0.05, ^**^p < 0.01). Plasma, *p* = 0.0041; kidney, *p* = 0.0086; brain, *p* = 0.0070; pancreas, *p* = 0.0288; gonads, *p* = 0.0445; femur, *p* = 0.0011.

Interestingly, major differences between the radioactivity uptakes in various organs were found for [^18^F]**1** and [^18^F]**2**. Figure 4 shows all organs (plus the blood and liver) in which significant differences in radioactivity uptake were found at 60 min after injection of the two radiotracers. The measured radioactivity was higher after application of [^18^F]**1** in those organs expressing CB_2_R natively [18] in comparison to the data obtained after injection of [^18^F]**2**. This relation is reversed in those organs associated to excretion. The cause of this is probably related to the much faster metabolism of [^18^F]**2** and its radiometabolites compared to that of [^18^F]**1**, which is in agreement with the observation of significantly lower accumulation of radiolabelled compounds in the plasma and kidney.

The significant differences in the pancreas and femur can be considered as a further indicator of different metabolic processes of [^18^F]**1** and [^18^F]**2** leading to different radiometabolites with diverse organ distribution patterns. Thus, the activity measured in the *ex vivo* biodistribution study is largely determined by metabolites bearing the ^18^F label.

### Metabolism of [^18^F]1 and [^18^F]2 in mice

**Figure 5:**
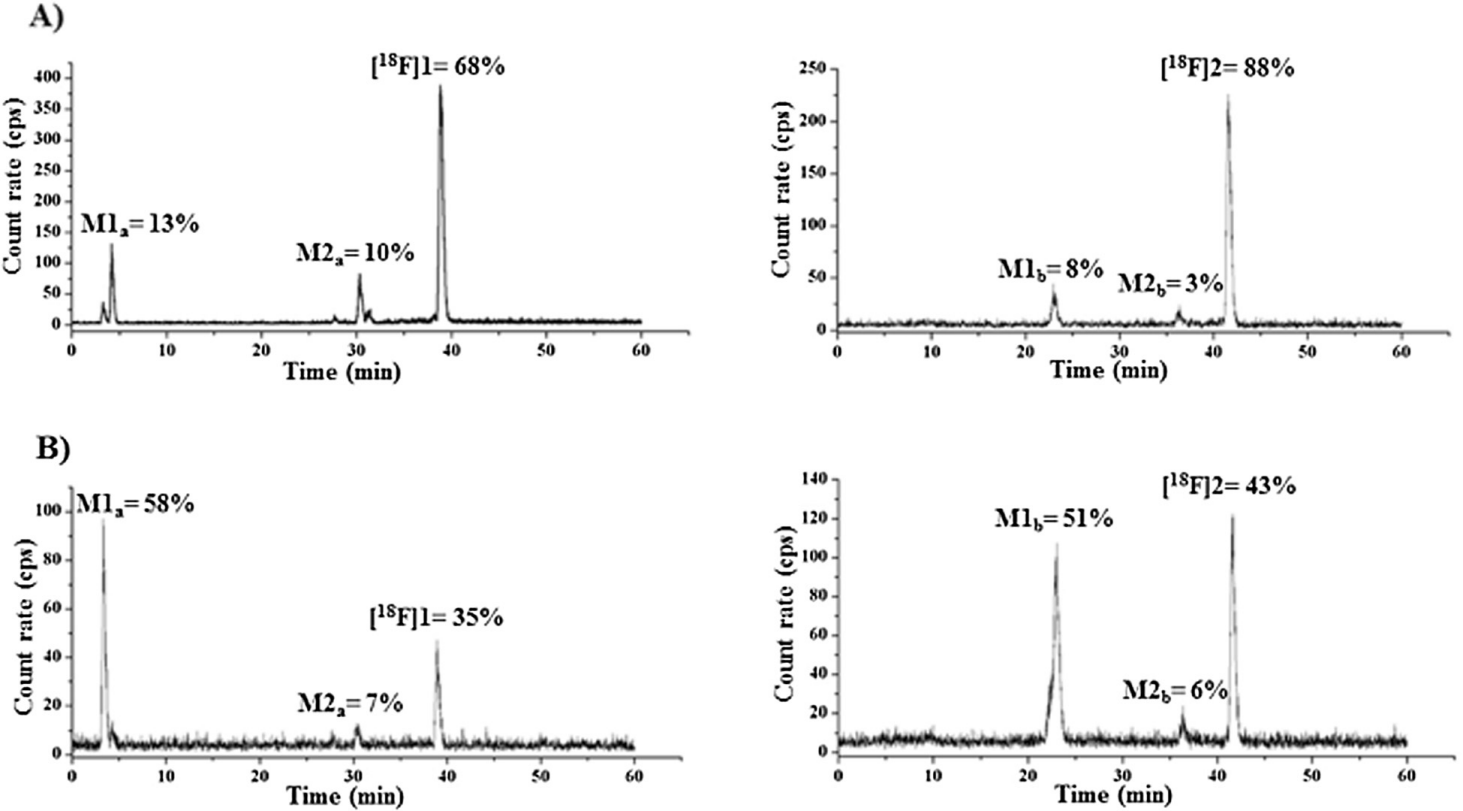
**Analytical radio-HPLC profile of acetonitrile extracts of brain homogenates. (A)** At 30 min p.i. and **(B)** 60 min p.i. Conditions: Multospher 120 RP 18 AQ, 5 μm, 250 × 4.6 mm. Solvents: A: 10% MeCN/20 mM NH_4_OAc, B: 90% MeCN/20 mM NH_4_OAc. Gradient elution (A%): 0 to 10 min, 100%; 10 to 40 min, gradient from 100% to 0%; 40 to 45 min, 0%; 45 to 50 min, 100% at a flow rate of 1 mL/min.

Typical HPLC radiochromatograms of brain extracts are shown in Figure 5. In general, radio-TLC analytics of plasma, urine, and brain samples obtained at 30 and 60 min p.i. of [^18^F]**1** or [^18^F]**2** are consistent with the data obtained by radio-HPLC. As shown in Table 4, unmetabolized [^18^F]**1** accounted for 60% of the recovered activity in plasma samples at 30 min p.i., while 36% of the total radioactivity can be addressed to a more hydrophilic metabolite **M1**_**a**_. The amount of intact radiotracer [^18^F]**1** is further reduced at 60 min p.i. to 7% with a concomitant increase of **M1**_**a**_ to 92%.

**Table 4:** Relative percentages of intact tracer and radiometabolites in plasma samples

**Compound**	***t***_**R **_**(min)**^**a**^	**Plasma**	
		**30 min p.i. (%)**	**60 min p.i. (%)**
[^18^F]**1**	38.8	60	7
**M1**_**a**_	4.2	36	92
**M2**_**a**_	30.2	3	1
[^18^F]**2**	41.7	2	2
**M1**_**b**_	22.9	95	88
**M2**_**b**_	36.4	≤1	≤1

At 30 and 60 min p.i. of [^18^F]**1** and [^18^F]**2**. ^a^Radio-HPLC (separation conditions, see ‘Experimental’ section).

Radiochromatograms of brain samples at 30 min p.i. shown in Figure 5 revealed that unmetabolized radiotracer corresponded to 68% and 88% of the total activity after administration of [^18^F]**1** and [^18^F]**2**, respectively. Paralleled by the constantly increasing accumulation of radiometabolites in the brain, these values decreased to 35% and 43%, respectively, at 60 min p.i. In particular, these radiometabolites account for 13% (**M1**_**a**_) and 10% **(M2**_**a**_) as well as 8% (**M1**_**b**_) and 3% (**M2**_**b**_) of total activity at 30 min p.i. of [^18^F]**1** and [^18^F]**2**, respectively, with the main radiometabolites **M1**_**a**_ and **M1**_**b**_ reaching values of 58% and 51% at 60 min p.i., respectively. The radiometabolite **M2**_**a**_ of [^18^F]**1** corresponds most likely to the amine, which is formed by enzymatic hydrolysis of the amide bond in the compound.

**Scheme 5:**
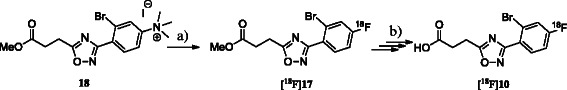
Radiosynthesis and metabolism of [^18^F]17 **Radiosynthesis and metabolism of [**^**18**^**F]17. (a)** Radiolabelling with K[^18^F]F-K_2.2.2_-carbonate complex (5.6 mg, 15 μmol), 2 mg of **18**, MeCN, 82°C, 10 min; **(b)***in vivo* metabolism.

The total amount of activity measured in, e.g. the plasma at particular times p.i. also depends on the kinetics of the excretion of radiotracer/radiometabolites. For [^18^F]**2**, this is more pronounced than for [^18^F]**1** with unmetabolized [^18^F]**2** accounting for only 2% of the total radioactivity in the plasma at 30 min p.i. At this time, more than 90% of the recovered activity in plasma samples for [^18^F]**2** consisted of the single radiometabolite **M1**_**b**_. As this value decreases only slightly up to 60 min p.i. (88%), **M1**_**b**_ can be assumed as a rather metabolically stable compound. This radiometabolite of [^18^F]**2** probably corresponds to the *N*-aryl-oxadiazolyl-propanoic acid resulting from enzymatic hydrolysis of the amide bond of **2**[40]. To prove this assumption, the trimethylammonium salt **18** was synthesized as the precursor of this metabolite. In order introduce ^18^F into the molecule, the carbon acid moiety was protected as a methyl ester resulting in the labelled compound [^18^F]**17** (Scheme 5). It was expected that after injection of this compound in mice, the ester will be converted into the free acid [^18^F]**10**. Those metabolic pathways are common for drug inactivation and excretion [41] as well as for the prodrug concept providing pharmacologically active metabolites *in vivo* from pharmacologically inactive compounds [42]. After intravenous injection of [^18^F]**17** in CD1 mice, samples of the plasma, brain, and spleen were analyzed at 30 and 60 min p.i. (Table 5).

**Table 5:** **[**^**18**^**F]10 uptake in female CD1mice after intravenous administration of 100 to 200 kBq [**^**18**^**F]17**

**Sample**	**30 min p.i. (% ID/g)**	**60 min p.i. (% ID/g)**
Plasma	18.21	15.38
Brain	0.28	0.29
Spleen	1.35	1.31

**Figure 6:**
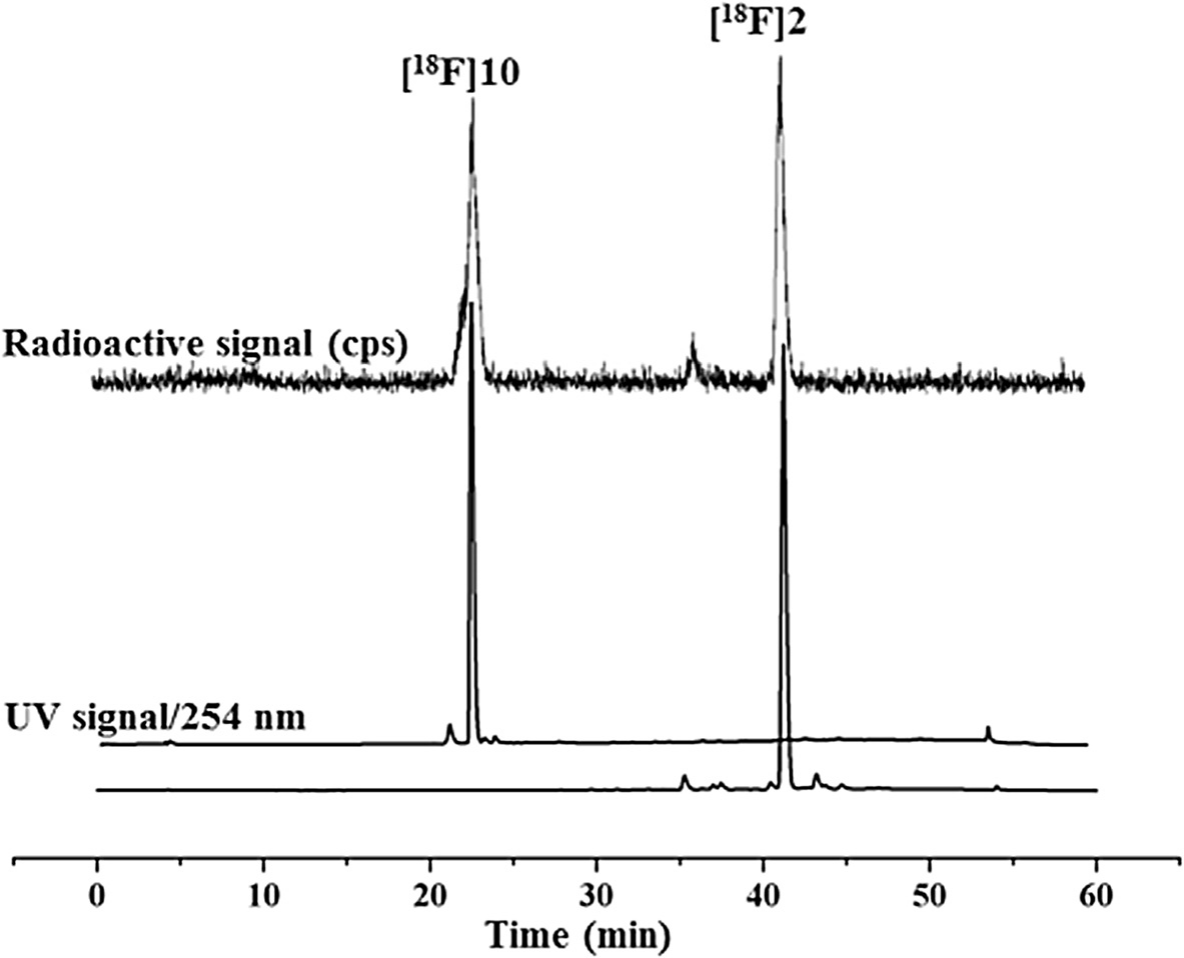
**Analytical radio-HPLC profile of [**^**18**^**F]2 at 60 min p.i spiked with the reference 2 and 10 (M1**_**b**_**).** Conditions: Multospher 120 RP 18 AQ, 5 μm, 250 × 4.6 mm. Solvents: A: 10% MeCN/20 mM NH_4_OAc, B: 90% MeCN/20 mM NH_4_OAc.Gradient elution (A%): 0 to 10 min, 100%; 10 to 40 min, gradient from 100% to 0%; 40 to 45 min, 0%; 45 to 50 min, 100% at a flow rate of 1 mL/min.

As expected, the methylester [^18^F]**17** (*t*_R_ = 36 min) is completely converted to free acid [^18^F]**10** (*t*_R_ = 22 min) in the plasma samples already at 30 min p.i. (Scheme 5). Radio-HPLC analysis of brain samples at 60 min p.i., spiked with the reference compounds **2** and **10**, confirmed that the radiometabolite **M1**_**b**_ co-elutes with compound **10** (Figure 6).

**Figure 7:**
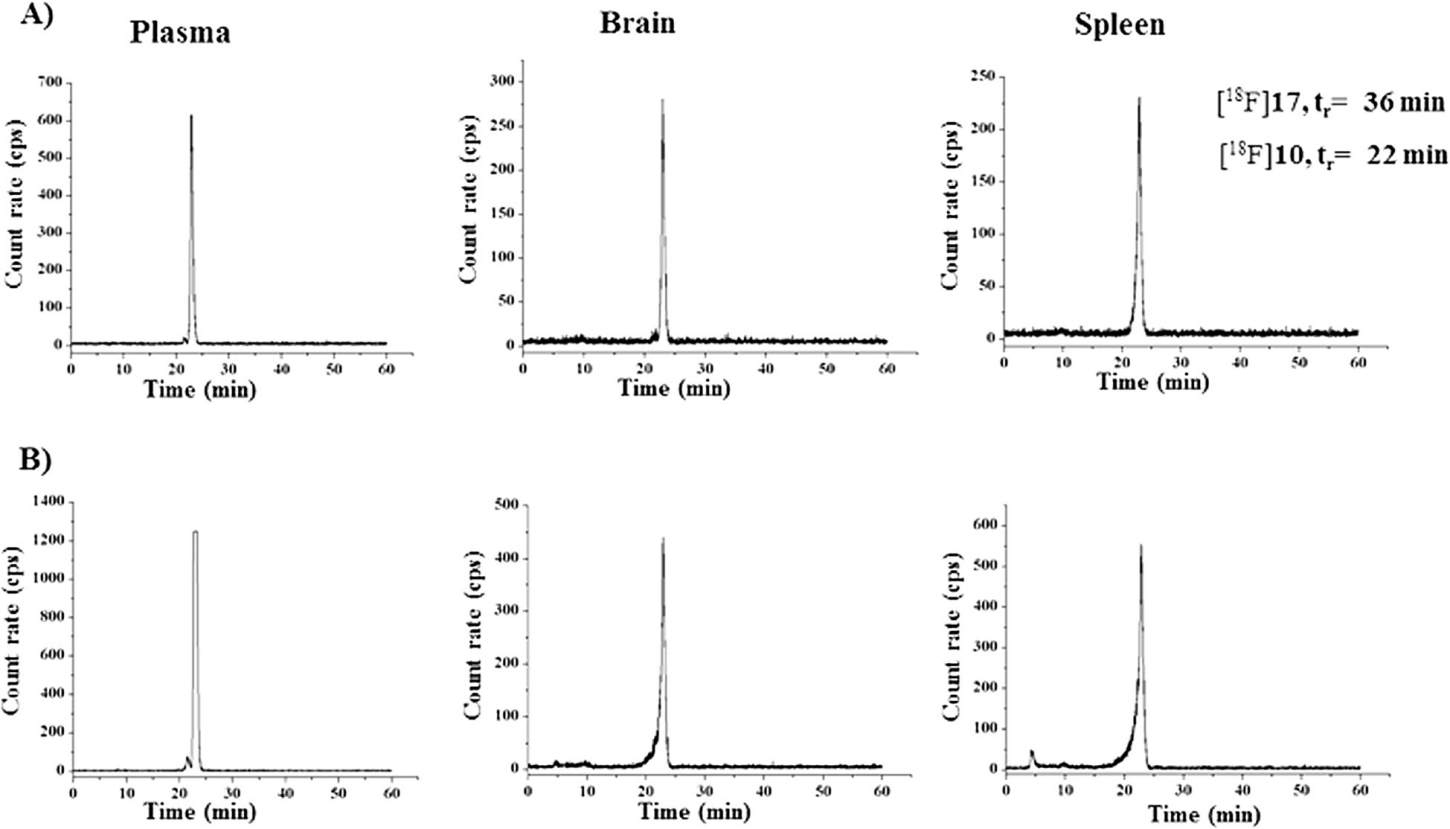
***In vivo *metabolism conversion of [**^**18**^**F]17 to [**^**18**^**F]10.** Analytical radio-HPLC profiles of the *in vivo* metabolism conversion of [^18^F]**17** (*t*_R_ = 36 min) to [^18^F]**10** (*t*_R_ = 22 min, Scheme 5b) in plasma, brain, and spleen samples. **(A)** At 30 min p.i. and **(B)** at 60 min p.i. Conditions: Multospher 120 RP 18 AQ, 5 μm, 250 × 4.6 mm. Solvents: A: 10% MeCN/20 mM NH_4_OAc, B: 90% MeCN/20 mM NH_4_OAc.Gradient elution (A%): 0 to 10 min, 100%; 10 to 40 min, gradient from 100% to 0%; 40 to 45 min, 0%; 45 to 50 min, 100% at a flow rate of 1 mL/min.

Furthermore, in combination with the time-activity data presented in Table 5, this result indicates that the main radiometabolite **M1**_**b**_ of [^18^F]**2** derived from peripheral metabolism ([^18^F]**10**) penetrates the blood–brain barrier (BBB). In addition, analyses of brain and spleen sample data revealed that no further metabolic transformation of [^18^F]**10** could be observed up to 60 min p.i. as shown in Figure 7.

*In vitro* investigation of the affinity of **10** towards both hCB_2_R and hCB_1_R revealed no specific binding (*K*_i_ > 1 μM). Altogether, these data suggest that non-target binding radiometabolites of [^18^F]**1** and [^18^F]**2** account for the vast majority of *ex vivo* activity measured in the spleen and other organs known to express CB_2_R. This corresponds very well with the insignificant displacement of activity after administration of [^18^F]**1** and [^18^F]**2** by the CB_2_R-specific SR144528. Hence, radiometabolites generated in the periphery penetrate the BBB leading to a pronounced accumulation of the main radiometabolites in the brain, hampering the application of [^18^F]**1** and [^18^F]**2** for imaging of CB_2_R in the brain.

## Experimental

### Materials and general procedures

Unless otherwise noted, moisture-sensitive reactions were performed under dry nitrogen or argon. All chemicals and reagents were purchased from commercially available sources and used without further purification unless otherwise specified. Tetrahydrofuran (THF) was dried with sodium/benzophenone and was freshly distilled before use. For the TLC, silica gel 60 F254 plates (Merck KGaA, Darmstadt, Germany) were used. For the flash chromatography (fc), silica gel 60, 40 to 64 μm, (Merck) was used. Room temperature (rt) was 20°C. Melting point was determined using Stuart™ Melting point apparatus SMP3 (Bibby Scientific Ltd, Staffordshire, UK), uncorrected. Mass spectrometry (MS) was done using MAT GCQ (Thermo Finnigan MAT GmbH, Bremen, Germany). ^1^H, ^13^C, and ^19^F NMR spectra were recorded on VARIAN GEMINI 2000 (200 MHz for ^1^H NMR, 50 MHz for ^13^C NMR, 188 MHz for ^19^F NMR), VARIAN MERCURY plus (300 MHz for ^1^H NMR, 75 MHz for ^13^C NMR, 228 MHz for ^19^F NMR), and VARIAN MERCURY plus and BRUKER DRX-400 (400 MHz for ^1^H NMR, 100 MHz for ^13^C NMR, 367 MHz for ^19^F NMR); δ (in ppm)was related to tetramethylsilane and trichloro-fluoro-methane (CF_3_Cl), respectively; coupling constants (*J*) were given with 0.1-Hz resolution. Multiplicities of NMR signals are indicated as follows: s (singlet), d (doublet), t (triplet), m (multiplet), dd (doublet of doublets), and dt (doublet of triplets). HPLC method for determination of the product purity was done with Merck Hitachi Equipment; L-7400 UV detector, L-7200 autosampler, L-7100 pump, and L-7614 degasser were used. The following conditions were used in the experiment: column, LiChrospher® 60 RP-select B, 5 μm, 250 × 4.0 mm; flow rate, 1 mL/min; injection volume, 5.0 μL; detection at *λ* = 210 nm. Solvents: A: water with 0.05% (*v*/*v*) trifluoroacetic acid, B: MeCN with 0.05% (*v*/*v*) trifluoroacetic acid. Gradient elution (A%): 0 to 4 min, 90%; 4 to 29 min, gradient from 90% to 0%; 29 to 31 min, 0%; 31 to 31.5 min, gradient from 0% to 90%; 31.5 to 40 min, 90%.

For radiochemistry, TLC was performed on silica gel pre-coated plates (Polygram, SIL G/UV254) with petroleum ether/ethyl acetate/ammonia (2:1:0.1, *v*/*v*/*v*), and the spots were visualized using UV light at 254 nm. Radio-TLC was recorded using a BAS-1800 II system Bioimaging Analyzer (Fuji Photo Film, Co. Ltd., Tokyo, Japan), and images were evaluated with AIDA 2.31 software (raytest Isotopenmessgeräte GmbH, Straubenhardt, Germany). Purification and isolation of the radiotracers were conducted on a semi-preparative radio-HPLC consisting of a S1021 pump (Sykam GmbH, Eresing, Germany), UV detector (WellChrom Filter-Photometer K-2001; Wissenschaftl. Gerätebau Dr. Ing. Herbert Knauer GmbH, Berlin, Germany), NaI(Tl) counter, and data acquisition by an automated system (Nina, Nuclear Interface, GE Medical Systems, Munich, Germany) on a Reprosil Gold C18, 10 μm, 150 × 4.6 mm, eluent: 58% MeCN/10 mM NH_4_OAc aq. at a flow rate of 2.5 mL/min, and Multospher 120 RP-18 AQ, 5 μm, 150 × 10 mm, eluent: 60% MeCN/20 mM NH_4_OAc aq. at a flow rate of 2.0 mL/min, respectively for [^18^F]**1** (*t*_R_ = 35.3 min) and [^18^F]**2** (*t*_R_ = 35.6 min). For the *in vivo* pharmacokinetic metabolite evaluation of [^18^F]**17** (*t*_R_ = 19.5 min) semi-preparative isolation was performed on a Multospher 120 RP 18 AQ, 5 μm, 150 × 10 mm, eluent: 56% MeCN/20 mM NH_4_OAc aq. at a flow rate of 2.0 mL/min. Analytical chromatographic separations were performed on a JASCO LC-2000 system (JASCO Labor- und Datentechnik GmbH, Gross-Umstadt, Germany), incorporating a PU-2080Plus pump, AS-2055Plus auto injector (100 μL sample loop) and a UV-2070Plus UV detector (monitoring at 254 nm). All analytical radio-HPLC analyses were performed using a gradient mode on a Multospher 120 RP18 AQ, 5 μm, 250 × 4.6 m. Solvents: A: 10% MeCN/20 mM NH_4_OAc, B: 90% MeCN/20 mM NH_4_OAc.Gradient elution (A%): 0 to10 min, 100%; 10 to 40 min, gradient from 100% to 0%; 40 to 45 min, 0%; 45 to 50 min, 100% at a flow rate of 1 mL/min.

#### (Z)-2-Bromo-N′-hydroxy-4-nitrobenzimidamide (5) and 2-bromo-4-nitrobenzamide (7)

To a solution of 2-bromo-4-nitrobenzonitrile **3** (100 mg, 0.44 mmol, 1 eq.) in 5 mL MeOH under argon, Na_2_CO_3_ (56 mg, 0.52 mmol, 1.2 eq.) and NH_2_OH-HCl (76 mg, 1.1 mmol, 2.5 eq.) were added at rt, and the reaction mixture was refluxed for 15 h. Upon cooling to rt, 10 mL of H_2_O was added and the aqueous phase was washed three times with 10 mL ethyl acetate (EtOAc). The combined organic extracts were dried over sodium sulfate and filtered. Upon evaporation of the solvent, column chromatography was performed (silica, EtOAc/isohexane (IH) 2:8) to give **5** (62 mg, 0.24 mmol, 55%) and **7** (41 mg, 0.17 mmol, 32%) as colorless solids. **5**: ^1^H NMR (400 MHz, DMSO-*d*_6_): *δ* (ppm) = 9.70 (s, 1H), 8.41 (d, *J* = 2.3 Hz, 1H), 8.21 (dd, *J* = 8.4/2.3 Hz, 1H), 7.64 (d, *J* = 8.4 Hz, 1H), 5.99 (s, 2H), 5.99 (s, 2H); ^13^C NMR (100 MHz, DMSO-*d*_6_): *δ* (ppm) = 150.4, 147.8, 141.7, 132.2, 127.3, 122.5, 122.3; MS (ESI+): *m*/*z* (%) = 260/262 (100/100) [M+H]^+^. **7**: ^1^H NMR (400 MHz, CDCl_3_): *δ* (ppm) = 8.49 (d, *J* = 2.2 Hz, 1H), 8.24 (dd, *J* = 8.5, 2.2 Hz, 1H), 7.79 (d, *J* = 8.5 Hz, 1H); ^13^C NMR (100 MHz, CDCl_3_): *δ* (ppm) = 164.0, 130.6, 128.7, 122.7, 120.0; MS (ESI+): *m*/*z* (%) = 245/247 (100/100) [M+H]^+^.

#### (Z)-2-Bromo-4-fluoro-N′-hydroxybenzimidamide (6) and 2-bromo-4-fluorobenzamide (8)

Compounds **6** and **8** (pale yellow solids) were obtained under the same synthetic procedure described for compounds **5** and **7** with 2-bromo-1-fluoro-4-nitrobenzene **4** as starting reagent. **6** (yield 73%): ^1^H NMR (400 MHz, CDCl_3_): *δ* (ppm) = 7.46 (dd, *J* = 8.6, 6.0 Hz, 1H), 7.35 (dd, *J* = 8.2, 2.5 Hz, 1H), 7.07 (dt, *J* = 8.1, 4.4 Hz, 1H); ^13^C NMR (100 MHz, CDCl_3_): *δ* (ppm) = 161.7, 151.8, 132.6 (d, *J* = 8.8 Hz), 120.7 (d, *J* = 24.7 Hz), 115.1 (d, *J* = 19.1 Hz), 114.9 (d, *J* = 19.0 Hz); ^19^F NMR (376 MHz, CDCl_3_): *δ* (ppm) = −109.3 (dd, *J* = 14.1, 7.9 Hz); MS (ESI+): *m*/*z* (%) = 233/235 (100/100) [M+H]^+^. **8** (yield 5%): ^1^H NMR (400 MHz, CDCl_3_): *δ* (ppm) = 7.49 (dd, *J* = 8.6, 5.9 Hz, 1H), 7.27 (dd, *J* = 8.4, 2.3 Hz, 1H), 7.00 (td, *J* = 8.2, 2.4 Hz, 1H), 3.44 (s, 2H); ^13^C NMR (100 MHz, CDCl_3_): *δ* (ppm) = 169.7 (d, *J* = 5.6 Hz), 164.2, 161.7, 133.2, 130.9 (d, *J* = 9.0 Hz), 120.7 (d, *J* = 24.7 Hz), 119.9 (d, *J* = 9.8 Hz), 114.7 (d, *J* = 21.4 Hz); MS (ESI+): *m*/*z* (%) = 218/212 (100/100) [M+H]^+^.

#### 3-(3-(2-Bromo-4-nitrophenyl)-1,2,4-oxadiazol-5-yl)propanoic acid (9)

The (Z)-2-bromo-*N*′-hydroxy-4-nitrobenzene-1-carboximidamide **5** (100 mg, 0.38 mmol, 1 eq.) was dissolved in 1 mL of dimethylformamide (DMF) under inert atmosphere. Succinic anhydride (95 mg, 0.95 mmol, 2.5 eq.) and KF (6.7 mg, 0.11 mmol, 0.3 eq.) were added, and the reaction mixture was warmed up to 130°C for 30 min. Upon cooling, 20 mL of H_2_O was added and the solid was collected by filtration, washed with H_2_O, and dried to give **9** as a colorless solid (123 mg, 0.36 mmol, 95%) with a melting point (mp) = 127°C. ^1^H NMR (400 MHz, DMSO-*d*_6_): *δ* (ppm) = 8.57 (d, *J* = 2.3 Hz, 1H), 8.36 (dd, *J* = 8.6, 2.3 Hz, 1H), 8.07 (d, *J* = 8.6 Hz, 1H), 3.23 (t, *J* = 6.9 Hz, 2H), 2.83 (t, *J* = 6.9 Hz, 2H); ^13^C NMR (100 MHz, DMSO-*d*_6_): *δ* (ppm) = 178.8, 173.2, 166.0, 148.4, 133.4, 132.3, 128.5, 122.0, 121.7, 29.5, 21.3; MS (ESI+): *m*/*z* (%) = 342/344 (100/100) [M+H]^+^.

#### 3-(3-(2-Bromo-4-fluorophenyl)-1,2,4-oxadiazol-5-yl)propanoic acid (10)

Compound **10** (colorless solid) was obtained under the same synthetic procedure described for compound **9** with (Z)-2-bromo-4-fluoro-*N*′-hydroxybenzen-1-carboximidamide **6** as starting reagent (yield 95%, mp = 104°C). ^1^H NMR (400 MHz, DMSO-*d*_6_): *δ* (ppm) = 7.86 (dd, *J* = 8.7, 6.1 Hz, 1H), 7.82 (dd, *J* = 8.6, 2.6 Hz, 1H), 7.50 to 7.41 (m, 1H), 3.20 (t, *J* = 6.9 Hz, 2H), 2.81 (t, *J* = 6.9 Hz, 2H); ^13^C NMR (100 MHz, DMSO-*d*_6_): *δ* (ppm) = 179.2, 172.7, 164.1, 161.6, 133.6 (d, *J* = 9.4 Hz), 124.3 (d, *J* = 3.5 Hz), 122.1 (d, *J* = 10.1 Hz), 121.4 (d, *J* = 25.1 Hz), 115.5 (d, *J* = 21.6 Hz), 29.9, 21.64; MS (ESI+): *m*/*z* (%) = 315/317 (100/100) [M+H]^+^.

#### 3-(3-(2-Bromo-4-nitrophenyl)-1,2,4-oxadiazol-5-yl)-N-(9-ethyl-9H-carbazol-3-yl)propanamide (11)

To a solution of acid **9** (50 mg, 0.14 mmol, 1 eq.) and 9-ethyl-9H-carbazol-3-amine **12** (30.9 mg, 0.14 mmol, 1 eq.) in 5 mL dichloromethane (DCM), *N*,*N*′-diisopropylcarbodiimide (45 μL, 0.21 mmol, 1.5 eq.) was added at 0°C, and the reaction was stirred at rt until **9** and **12** were not visible by TLC control (silica, EtOAc/IH 1:1) (2 h). The reaction was diluted with 5 mL of DCM and washed once with 10 mL 0.5 m aq. HCl, 10 mL 0.5 n aq. NaOH and 10 mL aq. NaCl saturated solution. The organic phase was dried over sodium sulfate and filtered. Evaporation of the solvent afforded a brown solid which was recrystallized from THF to give **11** (63 mg, 0.11 mmol, 85%) as a white solid. ^1^H NMR (400 MHz, CDCl_3_): *δ* (ppm) = 8.57 (d, *J* = 2.0 Hz, 1H), 8.36 to 8.14 (m, 2H), 8.03 (d, *J* = 8 Hz, 2H), 7.62 to 7.13 (m, 4H), 4.35 (q, *J* = 7.3 Hz, 2H), 3.49 (t, *J* = 7.4 Hz, 2H), 3.04 (t, *J* = 7.4 Hz, 2H), 1.43 (t, J = 7.2 Hz, 3H); MS (ESI+): *m*/*z* (%) = 556/558 (100/100) [M+Na]^+^.

#### 3-(3-(4-Amino-2-bromophenyl)-1,2,4-oxadiazol-5-yl)-N-(9-ethyl-9H-carbazol-3-yl)propanamide (19)

To a solution of oxadiazole **11** (30 mg, 0.05 mmol, 1 eq.) and SnCl_2_-2H_2_O (63 mg, 0.28 mmol, 5 eq.) in 2 mL of EtOH, concentrated aq. HCl (37%) was added at rt. Upon completion (TLC, silica, EtOAc/IH 1:1), 10 mL of saturated aq. NaHCO_3_ solution was added, and the mixture was extracted three times with 10 mL of EtOAc. The combined organic extracts were dried over sodium sulfate and filtered. Evaporation of the solvent afforded **19** (17.6 mg, 0.04 mmol, 70%) as a brown solid which was used in the next step without further purification. ^1^H NMR (300 MHz, CDCl_3_): *δ* (ppm) = 8.17 (d, *J* = 1.9 Hz, 1H), 7.89 (d, *J* = 7.8 Hz, 1H), 7.47 (d, *J* = 8.4 Hz, 1H), 7.37 (dt, *J* = 9.1, 4.6 Hz, 1H), 7.34 to 7.14 (m, 3H), 7.09 to 6.99 (m, 1H), 6.94 (d, *J* = 2.2 Hz, 1H), 6.61 (dd, *J* = 8.4, 2.3 Hz, 1H), 4.19 (q, *J* = 7.2 Hz, 2H), 3.24 (t, *J* = 7.4 Hz, 2H), 2.86 (t, *J* = 7.4 Hz, 2H), 1.25 (t, *J* = 7.2 Hz, 3H); ^13^C NMR (75 MHz, CDCl_3_): *δ* (ppm) = 178.2, 170.6, 169.2, 167.5, 157.9, 140.4, 137.2, 133.0, 132.7, 129.7, 125.7, 122.8, 122.7, 120.5, 120.4, 119.4, 118.6, 114.5, 112.7, 108.5, 108.4, 37.5, 32.6, 22.4, 13.6; MS (ESI+): *m*/*z* (%) = 528/526 (100/100) [M+Na]^+^.

#### 3-(3-(2-Bromo-4-(dimethylamino)phenyl)-1,2,4-oxadiazol-5-yl)-N-(9-ethyl-9H-carbazol-3-yl)propanamide (20)

To a solution of paraformaldehyde (PFA) (7.2 mg, 0.24 mmol, 6 eq.) in 90 μL of H_2_O, concentrated H_2_SO_4_ (13 μL, 0.24 mmol, 6 eq.) was added, and the reaction was cooled in an ice bath. To the resulting solution, **19** (20 mg, 0.04 mmol, 1 eq.) in 1 mL THF was added, and the reaction mixture was stirred for 30 min at 0°C. Next, NaBH_4_ (granules, 9.1 mg, 0.24 mmol, 6 eq.) was added in one portion, and the reaction was stirred for a further 18 h at rt. The reaction was quenched by addition of a concentrated aqueous NaOH solution (pH = 10 to 12), and the resulting aqueous phase was washed with EtOAc. The organic phase was dried over sodium sulfate and filtered. Upon purification by column chromatography (silica, EtOAc/IH 3/7) a yellow-brown solid was obtained which was further purified by recrystallization from THF to give **20** as a colorless solid (11 mg, 0.12 mmol, 53%) with mp = 125°C. ^1^H NMR (400 MHz, DMSO-*d*_6_): *δ* (ppm) = 10.11 (s, 1H), 8.39 (s, 1H), 8.02 (d, *J* = 7.6 Hz, 1H), 7.66 (d, *J* = 8.9 Hz, 1H), 7.57 to 7.45 (m, 3H), 7.48 to 7.32 (m, 1H), 7.20 to 7.07 (m, 1H), 6.95 (d, *J* = 2.6 Hz, 1H), 6.77 (dd, *J* = 8.9, 2.6 Hz, 1H), 4.38 (q, *J* = 7.0 Hz, 2H), 3.28 (t, *J* = 7.0 Hz, 2H), 2.96 (t, *J* = 7.1 Hz, 2H), 2.95 (s, 6H), 1.27 (t, *J* = 7.1 Hz, 3H); ^13^C NMR (100 MHz, DMSO-*d*_6_): *δ* (ppm) = 178.4, 168.6, 167.0, 156.7, 152.1, 139.9, 136.1, 132.2, 131.1, 125.7, 122.0, 121.8, 120.1, 118.8, 118.5, 116.0, 113.4, 111.1, 110.7, 109.1, 108.9, 40.6, 36.9, 32.0, 21.7, 13.6; MS (ESI+): *m*/*z* (%) = 556/554 (100/100) [M+Na]^+^.

#### 3-Bromo-4-(5-(3-((9-ethyl-9H-carbazol-3-yl)amino)-3-oxopropyl)-1,2,4-oxadiazol-3-yl)-N,N,N-trimethylbenzenaminium iodide (13)

To a solution of dimethylamine **20** (53 mg, 0.1 mmol, 1 eq.) in 1 mL of dry DMF under argon, MeI (124 μL, 20 mmol, 20 eq.) was added at rt. The reaction was sealed and warmed up to 35°C. Additional MeI (124 μL, 20 mmol, 20 eq.) was added after 2 and 5 days, while the reaction temperature was maintained at 35°C. Upon solvent evaporation, the resulting thick mass was washed three times with 4 mL of EtOAc to remove trace impurities and unreacted starting material (**20**). Upon drying, 4 mL of H_2_O was added. The mixture was sonicated and placed in a fridge (+4°C) for 1 h. The solid was filtrated and rewashed with 4 mL of ice-cold H_2_O. The trimethylammonium salt **13** was obtained as a light brown solid (57 mg, 0.85 mmol, 85%) with mp = 109°C. ^1^H NMR (400 MHz,CD_3_CN): *δ* (ppm) = 8.71 (s, 1H), 8.41 (d, *J* = 1.9 Hz, 1H), 8.25 (d, *J* = 2.7 Hz, 1H), 8.08 (d, *J* = 7.8 Hz, 1H), 8.05 (d, *J* = 8.8 Hz, 1H), 7.93 (dd, *J* = 8.9, 2.8 Hz, 1H), 7.57 (dd, *J* = 8.7, 2.1 Hz, 1H), 7.49 (dt, *J* = 15.4, 8.2 Hz, 3H), 7.20 (ddd, *J* = 7.9, 6.9, 1.2 Hz, 1H), 4.40 (q, *J* = 7.2 Hz, 2H), 3.59 (s, 9H), 3.39 (t, *J* = 6.9 Hz, 2H), 3.05 (t, *J* = 6.9 Hz, 2H), 1.37 (t, *J* = 7.2 Hz, 3H); ^13^C NMR (101 MHz, CD_3_CN): *δ* (ppm) = 181.1, 169.8, 167.4, 149.2, 141.3, 137.7, 134.1, 131.7, 131.6, 127.5, 126.8, 123.9, 123.4, 121.2, 121.0, 120.0, 119.5, 112.6, 109.9, 109.8, 58.1 (3C), 38.2, 33.2, 22.9, 14.0; HRMS (ESI+): *m*/*z* (%) = 546.1531/548.1436 (100/100) [M]^+^.

#### 2-(9H-Carbazol-9-yl)ethanol (25)

Under N_2_, 9*H*-carbazole (**23**, 5.43 g, 32.5 mmol, 1.0 eq.) was dissolved in THF (55 mL) and cooled down to −78°C. *n*-Butyllithium (1.2 M in *n*-hexane, 32.4 mL, 38.9 mmol, 1.2 eq.) was added dropwise, and the mixture was stirred for 30 min at −78°C. Then, a solution of ethylene sulfate (4.51 g, 36.3 mmol, 1.1 eq.) in THF (15 mL) was added, and the mixture was stirred for 1 h at −78°C and 24 h at rt. H_2_O was added and the mixture was acidified to pH 1 with concentrated H_2_SO_4_and heated to reflux for 24 h. Afterwards, the solution was neutralized with 5 M NaOH, brine was added and the mixture was extracted with CH_2_Cl_2_. The organic layers were dried (Na_2_SO_4_) and concentrated under reduced pressure, and the residue was purified by fc (*d* = 8 cm, *l* = 12 cm, cyclohexane/EtOAc 65:35, *R*_f_ 0.42). Pale yellow solid, mp = 80°C, yield 4.66 g (68%). Purity (HPLC): 98.1% (*t*_R_ = 19.28 min). ^1^H NMR (400 MHz, DMSO-*d*_6_): *δ* (ppm) = 3.86 (quart, *J* = 5.9 Hz, 2H), 4.47 (t, *J* = 5.5 Hz, 2H), 4.99 (t, *J* = 5.0 Hz, 1H), 7.24 (t, *J* = 7.4 Hz, 2H), 7.48 (t, *J* = 7.6 Hz, 2H), 8.18 (d, *J* = 7.8 Hz, 2H); ^13^C NMR (CDCl_3_): *δ* (ppm) = 45.3, 59.6, 109.6 (2C), 118.6 (2C), 120.2 (2C), 122.2 (2C), 125.0 (2C), 140.5 (2C); exact mass (APCI): *m*/*z* = calculated for C_14_H_13_NOH 212.1069, found 212.1060.

#### 2-(3-Nitro-9H-carbazol-9-yl)ethanol (24)

9-(Hydroxyethyl)carbazole**25** (5.11 g, 24.2 mmol, 1 eq.) was dissolved in CH_2_Cl_2_ (100 mL) and cooled down to 5°C to10°C. Concentrated nitric acid (density 1.5 g/mL, 1.7 mL, 36.3 mmol, 1.5 eq.) was added dropwise under vigorous stirring. Stirring was continued at <10°C until the starting material was transformed completely. H_2_O (25 mL) was added and the reaction mixture was neutralized with NaHCO_3_. Subsequently, brine was added and the mixture was extracted with CHCl_3_. The organic layer was dried (Na_2_SO_4_) and concentrated under reduced pressure; the residue was supported on silica and purified by fc (*d* = 8 cm, *l* = 12 cm, cyclohexane/EtOAc 50:50, *R*_f_ 0.27). Pale yellow solid, mp = 230°C, yield 3.34 g (54%). ^1^H NMR (CDCl_3_): *δ* (ppm) = 4.13 (t, *J* = 5.3 Hz, 2H), 4.55 (t, *J* = 5,3 Hz, 2H), 7.38 (t, *J* = 8.0 Hz, 1H), 7.51 to 7.60 (m, 3H), 8.13 (d, *J* = 7.5 Hz, 1H), 8.39 (dd, *J* = 8.9/2.0 Hz, 1H), 9.03 (d, *J* = 2.2 Hz, 1H); ^13^C NMR (DMSO-*d*_6_): *δ* (ppm) = 39.4, 59.6, 110.2, 110.9, 117.3 (2C), 120.7, 121.3, 122.0, 122.3, 127.4, 139.9, 141.8, 144.1; exact mass (APCI): *m*/*z* = calculated for C_14_H_12_N_2_O_3_Na 279.0740, found 279.0740.

#### 2-(3-Amino-9H-carbazol-9-yl)ethanol-hydrochloride (26-HCl)

3-Nitro-9-(hydroxyethyl)carbazole (**24**, 500 mg, 1.95 mmol, 1.0 eq.) was dissolved in THF (10 mL/100 mg) and Pd/C (100 mg) was added. The reaction mixture was stirred for 24 h at rt under H_2_ (balloon). The catalyst was removed by filtration over Celite® and the filtrate was concentrated under reduced pressure. The residue was dissolved in diethyl ether. Under N_2_, HCl-Et_2_O (2 mol/L, 2 mL, 4 mmol, 2.0 eq.) was added drop wise under vigorous stirring to produce the respective HCl salt. The precipitate was filtered, washed with cold MeOH, and used without further purification. *R*_f_ 0.35 (cyclohexane/ethyl acetate 30:70). Colorless solid with mp = 230°C, yield 492 mg (96%). ^1^H NMR (**26**-HCl, DMSO-*d*_6_): *δ* (ppm) = 3.78 (t, *J* = 5.5 Hz, 2H), 4.47 (t, *J* = 5.5 Hz, 2H), 7.24 (t, *J* = 7.4 Hz, 1H), 7.45 (dd, *J* = 8.7/2.1 Hz, 1H), 7.50 (t, *J* = 7,7 Hz, 1H), 7.66 (d, *J* = 8.3 Hz, 1H), 7.74 (d, *J* = 8.7 Hz, 1H), 8.12 (d, *J* = 2.1 Hz, 1H), 8.18 (d, *J* = 7.7 Hz, 1H), 10.4 (s, 3H); ^13^C NMR (**26**-HCl, DMSO-*d*_6_): *δ* (ppm) = 45.5, 59.7, 110.2, 110.8, 114.8, 119.3, 120.6, 121.5, 122.3, 122.8, 126.7 (2C), 139.7, 141.2; exact mass (APCI): *m*/*z* = calculated for C_14_H_15_N_2_O 217.1179, found 217.1179.

#### 3-[3-(2-Bromo-4-fluorophenyl)-1,2,4-oxadiazol-5-yl]-N-[9-(2-hydroxyethyl)-9H-carbazol-3-yl]propanamide (14)

Compound **10** (400 mg, 1.3 mmol 1 eq.) was treated with COMU® (652 mg, 1.5 mmol 1.2 eq.) and triethylamine (0.54 mL, 3.9 mmol, 3.3 eq.) in DMF (15 mL) for 30 min at rt. The reaction mixture was cooled down to 0°C, and a solution of the 3-aminocarbazole hydrochloride **26** (222 mg, 0.9 mmol, 1.0 eq.) in DMF was added dropwise. The mixture was stirred for 24 h at <10°C. Then, H_2_O and brine were added, the mixture was extracted with CHCl_3_, the combined organic layers were dried (Na_2_SO_4_) and concentrated *in vacuo*, and the residue was purified by fc (*d* = 5 cm, *l* = 15 cm, cyclohexane/EtOAc 15:85, *R*_f_ 0.51 (EtOAc)). Colorless solid, mp = 164°C, yield 368 mg (80%). Purity (HPLC), 97.7% (*t*_R_ = 20.24 min). ^1^H NMR (DMSO-*d*_6_): *δ* (ppm) = 2.99 (t, *J* = 7.0 Hz, 2H), 3.35 (t, *J* = 7.1 Hz, 2H), 3.76 (q, *J* = 5.6 Hz, 2H), 4.40 (t, *J* = 5.5 Hz, 2H), 4.86 (t, *J* = 5.4 Hz, 1H), 7.15 (t, *J* = 7.4 Hz, 1H), 7.41 (t, *J* = 6.5 Hz, 1H), 7.46 (td, *J* = 8.5/2.6 Hz, 1H), 7.51 (dd, *J* = 8.8/1.7 Hz, 1H), 7.54 (d, *J* = 8.8 Hz, 1H,), 7.57 (d, *J* = 8.3 Hz, 1H), 7.83 (dd, *J* = 8.6/2.6 Hz, 1H), 7.89 (dd, *J* = 8.7/6.1 Hz, 1H), 8.02 (d, *J* = 7.8 Hz, 1H), 8.39 (s, 1H), 10.13 (s, 1H); ^13^C NMR (DMSO-*d*_6_): *δ* (ppm) = 21, 32.0, 45.3, 59.6, 109.5, 109.7, 110.9, 115.5 (d, *J* = 21.6 Hz,1C), 118.5, 118.7, 120.0, 121.4 (d, *J* = 24.9 Hz, 1C), 121.7, 122.0, 122.2 (d, *J* = 10.1 Hz, 1C), 124.5 (d, *J* = 3.4 Hz,1C), 125.6, 131.0, 133.6 (d, *J* = 9.3 Hz, 1C), 137.0, 140.9, 162.9 (d, *J* = 253.4 Hz, 1C), 166.4, 168.5, 179.6; exact mass (ESI): *m*/*z* = calculated for C_25_H_20_^79^BrFN_4_O_3_H 523.0776, found 523.0783.

#### 3-[3-(2-Bromo-4-fluorophenyl)-1,2,4-oxadiazol-5-yl]-N-(9-ethyl-9H-carbazol-3-yl)propanamide (2)

The synthesis of **2** is described in [29].

#### 3-[3-(2-Bromo-4-fluorophenyl)-1,2,4-oxadiazol-5-yl]-N-[9-(2-fluoroethyl)-9H-carbazol-3-yl]propanamide (1)

Under N_2_, XtalFluor-E® (263 mg, 1.2 mmol, 1.5 eq.) was suspended in CH_2_Cl_2_ (15 mL). Triethylamine trihydrofluoride (NEt_3_-3HF) (0.2 mL, 1.2 mmol, 1.5 eq.) and a solution of **14** (400 mg, 0.8 mmol, 1 eq.) in CH_2_Cl_2_ (20 mL) were added to the suspension via cannula at −78°C. The resulting mixture was warmed up to rt within 3 h. An aqueous solution of Na_2_CO_3_ (5% *m*/*m*) was added, and the reaction mixture was stirred for 15 min at rt. After the addition of brine, the mixture was extracted with CH_2_Cl_2_, the organic layer was dried (Na_2_SO_4_) and concentrated *in vacuo*, and the residue was purified by fc (*d* = 6 cm, *l* = 15 cm, cyclohexane/EtOAc 20:80, *R*_f_ = 0.53 (EtOAc)). The product (**1**) was recrystallized from ethyl acetate yielding 82 mg of colorless solid (yield 20%) with mp of 201°C. Purity (HPLC), 96.7% (*t*_R_ = 21.46 min). ^1^H NMR (CDCl_3_): *δ* (ppm) = 2.97 (t, *J* = 7.0 Hz, 2H), 3.39 (t, *J* = 7.0 Hz, 2H), 4.51 (dt, *J* = 24.2/5.1 Hz, 2H), 4.72 (dt, *J* = 46.8/5.1 Hz, 2H), 7.03 to 7.09 (m, 1H), 7.16 (t, *J* = 7.0 Hz, 1H)_,_ 7.27 (d, *J* = 8.6 Hz, 1H), 7.32 (d, *J* = 8.2 Hz, 2H), 7.36 to 7.43 (m, 3H), 7.56 (s, 1H), 7.76 (dd, *J* = 8.7/6.0 Hz, 1H), 7.97 (d, *J* = 7.8 Hz, 1H), 8.41 (d, *J* = 1.8 Hz); ^13^C NMR (DMSO-*d*_6_): δ (ppm) = 21.8, 32.0, 42.9 (d, *J* = 19.7 Hz), 82.6 (d, *J* = 167.9 Hz), 109.5, 109.6, 110.9, 115.5 (d, *J* = 21.7 Hz), 118.8, 118.9, 120.0, 121.4 (d, *J* = 25.1 Hz), 121.9, 122.1, 122.2 (d, *J* = 9.9 Hz), 124.5 (d, *J* = 3.4 Hz), 125.8, 131.4, 133.6 (d, *J* = 9.3 Hz), 136.7, 140.6, 162.9 (d, *J* = 253.2 Hz), 166.4, 168.5, 179.6; HRMS (APCI): *m*/*z* = calculated for C_25_H_19_^79^BrF_2_N_4_O_2_H 525.0732, found 525.0757.

#### [2-(3-{3-[3-(2-Bromo-4-fluorophenyl)-1,2,4-oxadiazol-5-yl]propanamido}-9H-carbazol-9-yl)ethyl] 4-methylbenzenesulfonate (15)

Under N_2_, compound **14** (250 mg, 0.5 mmol, 1 eq.), 4-dimethylaminopyridine (17.5 mg, 0.15 mmol, 0.3 eq.), and triethylamine (0.25 mL, 1.8 mmol, 3.6 eq.) were dissolved in THF (20 mL) and cooled down to 0°C. A solution of *p*-toluenesulfonyl chloride (183 mg, 1.0 mmol, 2 eq.) in THF (10 mL) was added dropwise. After 20 min, the mixture was warmed up to rt and stirred for 48 h. Subsequently, 1 M aq. NaOH (3 mL) was added and the layers were separated. The aqueous layer was extracted with CH_2_Cl_2_ and the organic layer was washed with brine. The combined organic layers were dried (Na_2_SO_4_) and concentrated *in vacuo*, and the residue was purified by fc (*d* = 5 cm, *l* = 10 cm, cyclohexane/EtOAc 40:60, *R*_f_ = 0.34 (cyclohexane/EtOAc 50:50)). The product (**15**) was recrystallized from CH_2_Cl_2_ yielding 197 mg (colorless solid, mp = 136°C, yield 61%). Purity (HPLC), 96.2% (*t*_R_ = 22.85 min). ^1^H NMR (CDCl_3_): *δ* (ppm) = 2.24 (s, 3H), 3.04 (t, *J* = 7.0 Hz, 2H), 3.47 (t, *J* = 7.0 Hz, 2H), 4.38 (t, *J* = 5.4 Hz, 2H), 4.49 (t, *J* = 5.3 Hz, 2H), 6.85 (d, *J* = 8.0 Hz, 2H), 7.15 (t, *J* = 7.4 Hz, 1H), 7.17 to 7.23 (m, 5H), 7.33 (dd, *J* = 8.7/1.9 Hz, 1H), 7.39 (t, *J* = 8.1 Hz, 1H), 7.46 (dd, *J* = 8.2/2.4 Hz, 1H), 7.74 (s, 1H), 7.83 (dd, *J* = 8.7/6.0 Hz, 1H), 7.94 (d, *J* = 7.8 Hz, 1H), 8.31 (d, *J* = 1.7 Hz, 1H); ^13^C NMR (DMSO-*d*_6_): *δ* (ppm) = 21.0, 21.8, 32.0, 41.3, 68.8, 109.2, 109.3, 110.8, 115.5 (d, *J* = 21.1 Hz, 1C), 118.5, 118.8, 119.9, 121.4 (d, *J* = 24.9 Hz, 1C), 122.0, 122.1 (d, *J* = 9.9 Hz, 1C), 122.2, 124.5 (d, *J* = 3.3 Hz, 1C), 125.7, 126.6 (2C), 129.5 (2C), 131.0, 131.3, 133.6 (d, *J* = 9.2 Hz, 1C), 136.3, 140.3, 144.4, 162.9 (d, *J* = 253.5 Hz, 1C), 166.6, 168.5, 179.7; HRMS (APCI): *m*/*z* = calculated for C_32_H_26_^79^BrFN_4_O_5_SH 677.0864, found 677.0858.

#### Methyl-3-(3-(2-bromo-4-nitrophenyl)-1,2,4-oxadiazol-5-yl)propanoate (21)

At −10°C, SOCl_2_ (71 μL, 0.77 mmol, 3.3 eq.) was dissolved in 0.5 mL MeOH and stirred for 10 min. Acid **9** (100 mg, 0.29 mmol, 1 eq.) was then added and the reaction was allowed to reach room temperature within 1 h. The reaction was stirred further for 1 h at rt and quenched by the addition of 5 mL saturated aq. NaHCO_3_ solution, and the product was extracted with EtOAc (three times with 5 mL). Evaporation of the solvent afforded **21** in a quantitative manner (104 mg, 0.29 mmol). ^1^H NMR (400 MHz, CDCl_3_): *δ* (ppm) = 8.59 (d, *J* = 2.2 Hz, 1H), 8.26 (dd, *J* = 8.6, 2.2 Hz, 1H), 8.06 (d, *J* = 8.6 Hz, 1H), 3.73 (s, 3H), 3.32 (t, *J* = 7.2 Hz, 2H), 2.97 (t, *J* = 7.2 Hz, 2H); ^13^C NMR (100 MHz, CDCl_3_): *δ* (ppm) = 178.8, 171.6, 166.7, 149.0, 134.1, 132.8, 129.4, 122.8, 122.2, 52.3, 30.2, 22.1; MS (ESI+): *m*/*z* (%) = 356/358 (100/100) [M+H]^+^.

#### Methyl-3-(3-(4-amino-2-bromophenyl)-1,2,4-oxadiazol-5-yl)propanoate (16)

To a solution of **21** (50 mg, 0.13 mmol, 1 eq.) in 2 mL EtOH, SnCl_2_-2H_2_O (149 mg, 0.66 mmol, 5 eq.) was added and the reaction was stirred for 2 h at rt. Saturated aq. NaHCO_3_ solution (5 mL) was added, and the mixture was extracted with EtOAc. The organic phase was dried over sodium sulfate and filtered. Upon solvent evaporation under reduced pressure, amine **16** was obtained as a brown solid (45 mg, 0.13 mmol, quant). ^1^H NMR (400 MHz, DMSO-*d*_6_): *δ* (ppm) = 7.45 (d, *J* = 8.5 Hz, 1H), 6.92 (d, *J* = 2.2 Hz, 1H), 6.64 (dd, *J* = 8.5, 2.2 Hz, 1H), 3.55 (s, 4H), 3.13 (dd, *J* = 7.6, 5.9 Hz, 2H), 2.93 – 2.74 (m, 2H); ^13^C NMR (100 MHz, DMSO-*d*_6_): *δ* (ppm) = 178.6, 172.8, 167.7, 152.5, 133.2, 122.4, 118.7, 114.1, 113.6, 52.4, 30.2, 22.0; MS (ESI+): *m*/*z* (%) = 348/350 (100/100) [M+Na]^+^.

#### Methyl-3-(3-(2-bromo-4-(dimethylamino)phenyl)-1,2,4-oxadiazol-5-yl)propanoate (22)

Compound **22** was synthesized as described for **20** from **16** in 52% yield as a brown solid. ^1^H NMR (300 MHz, CDCl_3_): *δ* (ppm) = 7.75 (d, *J* = 8.8 Hz, 1H), 7.00 (d, *J* = 2.6 Hz, 1H), 6.70 (dd, *J* = 8.9, 2.6 Hz, 1H), 3.72 (s, 3H), 3.25 (t, *J* = 7.4 Hz, 2H), 3.01 (s, 6H), 2.93 (t, *J* = 7.4 Hz, 2H); ^13^C NMR (75 MHz, CDCl_3_): *δ* (ppm) = 177.2, 171.9, 168.0, 152.0, 132.7, 123.1, 117.2, 115.2, 111.0, 52.3 (2C), 40.4, 30.5, 22.2; MS (ESI+): *m*/*z* (%) = 360/362 (100/100) [M+Na]^+^.

#### 3-Bromo-4-(5-(3-methoxy-3-oxopropyl)-1,2,4-oxadiazol-3-yl)-N,N,N-trimethylbenzenaminium iodide (18)

Compound **18** was synthesized as described for **13** from **22** in 90% yield as a brown solid (mp = 119°C). ^1^H NMR (300 MHz, CD_3_CN): *δ* (ppm) = 8.29 (d, *J* = 2.6 Hz, 1H), 8.04 (d, *J* = 8.8 Hz, 1H), 7.98 (dd, *J* = 8.9, 2.7 Hz, 1H), 3.68 (s, 3H), 3.63 (s, 9H), 3.29 (t, *J* = 6.9 Hz, 2H), 2.94 (t, *J* = 6.9 Hz, 2H); ^13^C NMR (75 MHz, CD_3_CN): *δ* (ppm) = 181.1, 173.4, 168.0, 134.7, 132.1, 128.2, 124.4, 121.8, 118.8, 58.8 (3C), 53.1, 31.3, 23.3; HRMS (ESI+): *m*/*z* (%) = 368.0616/370.0615 (100/100) [M]^+^.

#### Methyl-3-(3-(2-bromo-4-fluorophenyl)-1,2,4-oxadiazol-5-yl)propanoate (17)

Compound **17** was synthesized as described for **21** from **10** in a quantitative manner as a colorless solid (mp = 39°C). ^1^H NMR (400 MHz, CDCl_3_): *δ* (ppm) = 7.84 (dd, *J* = 8.7, 6.0 Hz, 1H), 7.47 (dd, *J* = 8.3, 2.5 Hz, 1H), 7.14 (ddd, *J* = 8.7, 7.7, 2.6 Hz, 1H), 3.73 (s, 3H), 3.29 (t, *J* = 7.3 Hz, 2H), 2.94 (t, *J* = 7.3 Hz, 2H); ^13^C NMR (100 MHz, CDCl_3_): *δ* (ppm) = 178.1, 171.7, 167.3, 164.7, 162.2, 133.4 (d, *J* = 9.1 Hz), 122.9 (d, *J* = 9.9 Hz), 121.7 (d, *J* = 24.7 Hz), 115.0 (d, *J* = 21.4 Hz), 52.2, 30.3, 22.1; ^19^ F NMR (376 MHz, CDCl_3_): *δ* (ppm) = −108.0 (dd, *J* = 14.1, 7.9 Hz); MS (ESI+): *m*/*z* (%) = 351/353 (100/100) [M+Na]^+^.

### Radiochemistry

No-carrier-added [^18^F]fluoride (*t*_1/2_ = 109.8 min) was produced via the [^18^O(p,n)^18^F] nuclear reaction by irradiation of a [^18^O]H_2_O target (Hyox 18 enriched water, Rotem Industries Ltd, Israel) on a Cyclone®18/9 (iba RadioPharma Solutions, Belgium) with a fixed energy proton beam using Nirta® [^18^F]fluoride XL target. Aqueous [^18^F]fluoride was dried by azeotropic distillation using MeCN in the presence of K_2_CO_3_ (1.78 mg, 12.9 mmol) and Kryptofix 2.2.2 (K_2.2.2_, 11.2 mg, 29.7 mmol), resulting in the reactive anhydrous K[^18^F]F-K_2.2.2_-carbonate complex. Optimization of the aliphatic and aromatic radiolabellings of tosylate **15** and trimethylammonium salt **13**, respectively, were performed by varying the amount of precursor for radiolabelling and reaction time under thermal heating (82°C) in MeCN (Scheme 1). Under optimized conditions, the reaction mixtures of [^18^F]**1** and [^18^F]**2** were diluted with water and directly applied to an isocratic semi-preparative RP-HPLC for isolation of the desired radiotracers (see ‘Experimental’ section). The collected fractions were analyzed by radio-TLC, and those with the highest radiochemical purity were combined, diluted with water, passed through a Sep-Pak C18 light cartridge (Waters, Milford, MA, USA), and eluted with 0.6 mL of diethyl ether (DEE). The elution of [^18^F]**1** and [^18^F]**2** was performed in polypropylene vials containing 75 to 100 μL of EtOH. For biological investigations, the solvent was evaporated to dryness under a gentle argon stream, and the desired radiotracers were formulated in a sterile isotonic solution containing 10% EtOH (*v*/*v*). The identities of [^18^F]**1** and [^18^F]**2** were verified by radio-HPLC of samples of the respective radiotracer spiked with the non-radioactive reference compound. Radiochemical and chemical purities were assessed by radio-TLC and analytical HPLC. Specific activities were calculated using the HPLC method described in [43]. For evaluation of radiometabolites, the radiosynthesis of the trimethylammonium salt **18** to afford [^18^F]**17** was performed following the same conditions as above stated for [^18^F]**1** and [^18^F]**2** (Scheme 5).

### Determination of lipophilicity (logD) and stability

The distribution coefficients of [^18^F]**1** and [^18^F]**2** were also calculated from RP-HPLC retention times in three different systems: (1) isocratic elution (60% MeCN/20 mM NH_4_OAc at a flow rate of 1 mL/min) and (2) gradient elution (starting from 10% MeCN/20 mM NH_4_OAc aq. for 10 min with a gradient to 90% MeCN/20 mM NH_4_OAc aq. over 30 min at a flow rate of 1 mL/min) on a Reprosil-Pur C18-AQ column (5 μm, 250 × 4.6 mm, Dr. Maisch HPLC GmbH, Ammerbruch-Entringen, Germany), and (3) isocratic elution (52.5% MeCN/20 mM NH_4_OAc at a flow rate of 1 mL/min) on a Prodigy 5 μm C8 (250 × 4 mm; Phenomenex Ltd, Aschaffenburg, Germany) using the reference compounds following the protocol according to the EU guideline 67/548/EWG [44].

*In vitro* radiochemical stabilities of [^18^F]**1** and [^18^F]**2** were investigated in 0.9% NaCl solution, Dulbecco’s phosphate buffer, 0.01 M Tris–HCl (pH 7.4 at 21°C), and EtOH at 40°C for 90 min. Samples were taken at 15, 30, 60, and 90 min after incubation and analyzed by radio-TLC and radio-HPLC.

### *In vitro* CB receptor affinity assay

For binding experiments, Chinese hamster ovary (CHO) cell lines stably transfected with human CB_1_R human and CB_2_R were used according to the procedures previously described [29]. Briefly, displacement of CB_1_R/CB_2_R-specific radioligand [^3^H]CP55,940 (6,438 GBq/mmol; PerkinElmer Life and Analytical Sciences GmbH, Rodgau, Germany; working concentration, 0.2 to 0.5 nM) by test compounds in the range of 0.1 nM to 10 μ was assessed, and IC_50_ values were estimated by non-linear regression (GraphPad Prism; version 3.0, GraphPad Software, Inc., San Diego, CA, USA). *K*_D_ values of 2.4 and 1.5 nM were previously determined for [^3^H]CP55,940 binding to hCB_1_R and hCB_2_R, respectively, and used for calculation of *K*_i_ values of the test compounds according to Cheng et al. [45]. The binding experiments were performed in triplicates, and data were given as mean values from independent experiments.

### *Ex vivo* biodistribution studies

Animals for *in vivo* studies were obtained from the Medizinisch-Experimentelles Zentrum, Universität Leipzig. All procedures that include animals were approved by the respective State Animal Care and Use Committee and conducted in accordance with the German Law for the Protection of Animal.

Female CD1 mice (10 to 12 weeks old, 20 to 25 g) received an injection of 300 to 400 kBq of [^18^F]**1** or [^18^F]**2** with specific activities of >450 GBq/μmol in 200 μL of 0.9% NaCl/10% EtOH into the tail vein. The animals were anesthetized (CO_2_/O_2_ mixture) for blood and urine sampling and euthanized by luxation of the cervical spine at 5, 30, and 60 min after injection (p.i.) (*n* = 2 to 5 per time). The organs of interest were removed and weighed, and the activities were measured by γ counting using a calibrated γ counter Wallac Wizard 1470 (Perkin Elmer Inc., Waltham, MA, USA). The percentage of injected dose per gram of wet tissue (% ID/g wet weight) was calculated.

To verify the specificity of [^18^F]**1** and [^18^F]**2** towards CB_2_R, blocking experiments were performed with pre-administration of the highly selective CB_2_R inverse agonist SR144528 [46] (3 mg/kg i.p. in 0.9% saline, 10 min before the injection of the radiotracer) at 60 min p.i. (*n* = 2 to 5). The unpaired two-tailed *t* test was used to compare the results between the groups. We considered differences to be significant at a *p* value <0.05.

### *Ex vivo* metabolite studies

[^18^F]**1** and [^18^F]**2** (100 to 150 MBq, 250 to 450 GBq/μmol in 150 μL NaCl 0.9%/10% EtOH) were injected via the tail vein in CD1 male mice (10 to 12 weeks old, 20 to 25 g). Blood and urine samples were obtained at 30 and 60 min p.i. (*n* = 3 per time point). Twofold extractions of plasma and brain samples (*n* = 3) were performed using ice-cold MeCN according to the standard protocol established in our group (see [47,48]). Briefly, plasma samples were obtained by centrifugation of the blood at 4,000×*g* at 4°C for 10 min, and brain samples were homogenized in ice-cold 50 mM Tris–HCl (pH = 7.4). The samples were vortexed, incubated on ice, and centrifuged at 10,000×*g* for 3 min. Supernatants were collected, and the precipitates were re-dissolved in ice-cold MeCN for the second extraction. The supernatants from the two extractions were combined, concentrated under a gentle argon stream at 65°C, and analyzed by radio-TLC and gradient analytical HPLC (see ‘Radiochemistry’ section). Aliquots from each extraction supernatant and the precipitates were also taken and quantified by γ counting (Wallac Wizard 1470, Perkin Elmer Inc., Waltham, MA, USA) along with the respective aliquots of intact plasma samples and brain homogenates. A moderate recovery of radioactivity was obtained from plasma samples and brain homogenates (60% to 70%). Radiometabolites of [^18^F]**17** in the plasma, spleen, and brain were assessed at 30 and 60 min p.i.

## Conclusions

In conclusion, *N*-aryl-oxadiazolyl-propionamides were successfully radiolabelled with ^18^F at different positions. Fluorine substitution at these positions did not affect affinity and specificity towards CB_2_R. However, the radiotracers investigated in this study undergo a fast metabolism *in vivo* with the main radiometabolites crossing the blood–brain barrier. Therefore, structural changes in the enzymatic cleavage sites of the evaluated candidates have to be performed to enhance their potential as CB_2_R PET imaging agents for the brain.

## Abbreviations

ALS: Amyotrophic lateral sclerosis; CB1R: Cannabinoid receptor type 1; CB2R: Cannabinoid receptor type 2; CHO: Chinese hamster ovary; COMU: (1-Cyano-2-ethoxy-2-oxoethylidenaminooxy)dimethylamino-morpholino-carbenium hexafluorophosphate; DCM: Dichloromethane; DEE: Diethyl ether; DIC: *N*,*N*′-diisopropylcarbodiimide; DMF: Dimethylformamide; EtOAc: Ethyl acetate; fc: Flash chromatography; GPCR: G protein-coupled receptors; ID: Injected dose; IH: Isohexane; MeI: Methyl iodide; mp: Melting point; NCA: No-carrier added; p.i.: Post injection; PET: Positron emission tomography; PFA: Paraformaldehyde; RCY: Radiochemical yields; Succ anh: Succinic anhydride; THF: Tetrahydrofuran; TLC: Thin-layer chromatography; XtalFluor-E: Diethylaminodifluorosulfonium tetrafluoroborate; 4-DMAP: 4-dimethylaminopyridine.

## Competing interests

The authors declare that they have no competing interests.

## Supplementary Material

Additional file 1 **Supplemental information.** In the additional file, data of the blocking studies (% blocking, *p* values) are compiled. Click here for file
