# Supplementary material for: Radiofluorination and biological evaluation of N-aryl-oxadiazolyl-propionamides as potential radioligands for PET imaging of cannabinoid CB2 receptors
Source: Org Med Chem Lett. 2013 Sep 24;3:11. doi: 10.1186/2191-2858-3-11 (PMC3856494; doi:10.1186/2191-2858-3-11)
Supplement: Additional file 1 — Supplemental information. In the additional file, data of the blocking studies (% blocking, p values) are compiled. [file 2191-2858-3-11-S1.doc]

# Supplemental Information

# Radiofluorination and Biological Evaluation of *N*-aryl-oxadiazolyl-propionamides as Potential Radioligands for PET Imaging of Cannabinoid CB2 Receptors

Rodrigo Teodoro,^1#^ Rareş-Petru Moldovan,^1#^ Corinna Lueg,^2#^ Robert Günther,^1^ Cornelius K. Donat,^1^ Friedrich-Alexander Ludwig,^1^ Steffen Fischer,^1^ Winnie Deuther-Conrad,^1^ Bernhard Wünsch,^2^ Peter Brust^2*^

* Corresponding author
Email: p.brust@hzdr.de

^1^ Department of Neuroradiopharmaceuticals, Institute of Radiopharmaceutical Cancer Research, Research Site Leipzig, Helmholtz-Zentrum Dresden-Rossendorf e.V., Permoserstr. 15, D-04318 Leipzig, Germany

^2^ Department of Pharmaceutical and Medicinal Chemistry, University of Münster, Münster, Corrensstraße 58-62, D-48149 Münster, Germany

# These authors contributed equally to the work.

**Table** **S1** **Radioactivity uptake (in %ID/g) under blocking conditions in major organs of female CD1-mice after intravenous administration of 300–400 kBq radiotracer.** Blocking was performed with pre-administration of SR144528 (3 mg kg^-1^ intraperitoneal). Values are means±SD.

|  | [^18^F]**1** |  |  |  |  | [^18^F]**2** |  |  |  |
| --- | --- | --- | --- | --- | --- | --- | --- | --- | --- |
| Organ | 60 min^a^ |  | % control | p-value^b^ |  | 60 min^c^ |  | % control | p-value^b^ |
| Blood | 1.43±0.34 |  | 74 | 0.145 |  | 3.40±0.21 |  | 136 | 0.030 |
| Plasma | 1.87±0.41 |  | 75 | 0.147 |  | 5.78±0.07 |  | 111 | 0.256 |
| Brain | 0.66±0.11 |  | 72 | 0.060 |  | 0.28±0.03 |  | 108 | 0.665 |
| Heart | 1.49±0.29 |  | 90 | 0.437 |  | 1.53±0.13 |  | 106 | 0.576 |
| Lung | 1.47±0.29 |  | 83 | 0.376 |  | 2.31±0.19 |  | 92 | 0.592 |
| Stomach | 1.84±1.28 |  | 68 | 0.324 |  | 3.24±2.10 |  | 146 | 0.455 |
| S. intestine | 19.39±5.53 |  | 88 | 0.545 |  | 29.64±5.10 |  | 109 | 0.514 |
| L. intestine | 1.63±0.64 |  | 106 | 0.788 |  | 1.78±0.23 |  | 96 | 0.770 |
| Liver | 4.22±0.96 |  | 80 | 0.136 |  | 10.51±1.10 |  | 168 | 0.005 |
| Kidney | 1.74±0.48 |  | 106 | 0.759 |  | 4.91±1.51 |  | 124 | 0.446 |
| Bladder | 5.07±3.84 |  | 155 | 0.368 |  | 1.77±0.16 |  | 122 | 0.407 |
| Spleen | 0.98±0.21 |  | 65 | 0.075 |  | 0.74±0.10 |  | 110 | 0.487 |
| Thymus | 1.50±0.35 |  | 79 | 0.367 |  | 1.77±0.14 |  | 171 | 0.002 |
| Pancreas | 1.03±0.26 |  | 91 | 0.432 |  | 1.02±0.11 |  | 125 | 0.210 |
| Adrenals | 3.00±0.64 |  | 95 | 0.787 |  | 3.34±0.15 |  | 137 | 0.099 |
| Gonads | 1.19±0.30 |  | 89 | 0.580 |  | 1.97±0.39 |  | 86 | 0.459 |
| Muscle | 0.93±0.23 |  | 104 | 0.784 |  | 0.70±0.06 |  | 99 | 0.960 |
| Femur | 1.49±0.45 |  | 81 | 0.200 |  | 0.61±0.09 |  | 106 | 0.713 |

^a^ n=3. ^b^ unpaired two-tailed t-test significant values with p<0.05. ^c^ n=6.


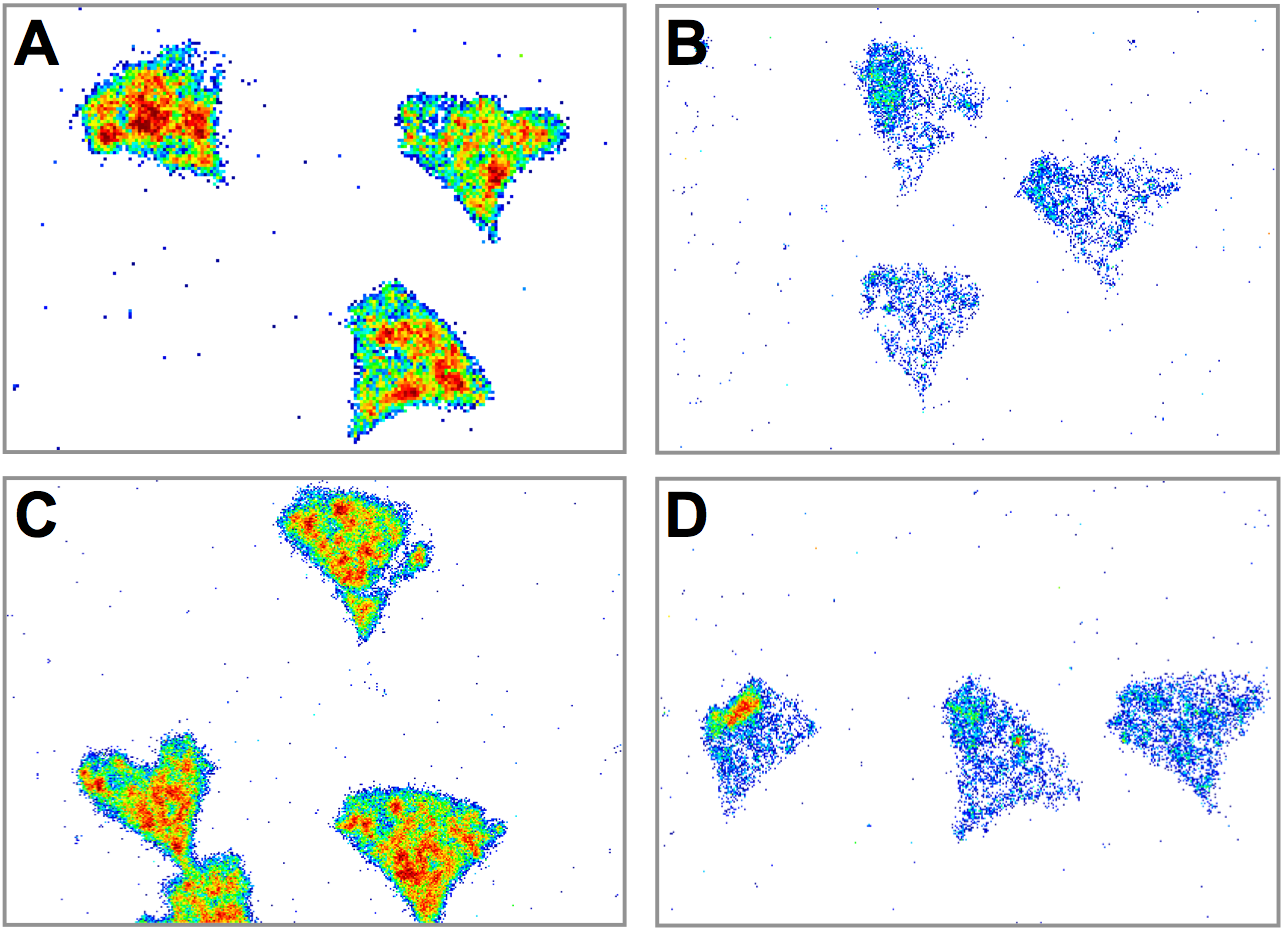


Figure S1 Autoradiogram of representative coronal sections of rat spleen tissue (SPRD rats, 10 weeks, slices 12 µm). (A) Incubated with [^18^F]1 (1 MBq/ml) and incubations with [^18^F]1 and (B) 10 µM 1, (C) 10 μM SR141716A (CB_1_R selective), (D) SR144528 (CB_2_R selective).
